# Nonanebis(peroxoic acid): a stable peracid for oxidative bromination of aminoanthracene-9,10-dione

**DOI:** 10.3762/bjoc.10.90

**Published:** 2014-04-24

**Authors:** Vilas Venunath Patil, Ganapati Subray Shankarling

**Affiliations:** 1 Department of Dyestuff Technology, Institute of Chemical Technology, N. P. Marg, Matunga, Mumbai - 400019, India. Tel.: 91-22-33612708, Fax: 91-22-33611020

**Keywords:** aminoanthracene-9,10-dione, benzanthrone, KBr, nonanebis(peroxoic acid), oxidative bromination

## Abstract

A new protocol for the oxidative bromination of aminoanthracene-9,10-dione, which is highly deactivated towards the electrophilic substitution is investigated. The peracid, nonanebis(peroxoic acid), possesses advantages such as better stability at room temperature, it is easy to prepare and non-shock sensitiv as compared to the conventional peracids. The present protocol has a broad scope for the bromination of various substituted and unsubstituted aminoanthracene-9,10-diones.

## Introduction

The brominated aminoanthracene-9,10-dione derivatives and benzanthrone are widely used as intermediates for pharmaceuticals [1–3], for medicinal applications [4], as dyes [5–9] and in Hg^2+^ ion sensors [10]. Although these bromo derivatives have a wide range of applications, it is difficult to carry out their bromination as the two carbonyl groups of aminoanthracene-9,10-dione deactivate the ring towards electrophilic substitution [11]. Generally, the bromination of aminoanthracene-9,10-dione is carried out in sulfuric acid [12–15] (20 to 98%) and nitrobenzene [16–17] at a high temperature (80 to 120 °C) with or without a catalyst (like Cu or CuSO_4_) [18–21]. In all processes, hazardous molecular bromine is used as a bromine source [11–21]. Ghaieni et. al. [22–23] have reported bromination of aminoanthracene-9,10-dione in a mixture of conc sulfuric acid with chlorobenzene, glacial acetic acid, methanol and also with molecular iodine as a catalyst. These reactions have various disadvantages such as long reaction time (up to 24 h), high temperature (60 to 100 °C) and are accompanied by a mixture of mono- and dibromo products. The reaction performed in glacial acetic acid at 100 °C resulted in 76% of the desired dibromo product in 6 h, along with the starting amine and some byproducts. The long reaction time, drastic reaction conditions, isomer formation and their separation (which is a very tedious and difficult task), and reduced bromine atom economy make these approaches economically unattractive. Again, in all the above processes, molecular bromine is used as the bromine source which reduces the bromine atom economy by 50% [24], as the HBr generated in the reaction is not used for further bromination and also causes pollution of the environment. This problem can be resolved by employing an oxidant in the reaction, which re-oxidizes the HBr and utilizes up to 90–95% [25–26] of bromine. Various oxidants are used for this purpose [27–30]. Compared to these commonly used oxidants the use of peracids is likely to be limited due to their instability and the resulting storage issues. These peracids are either have to be prepared in situ or they require cold storage conditions [31]. Finding that there is a need to develop a stable peracid, we synthesized a stable, solid, aliphatic long chain peracid, nonanebis(peroxoic acid). This peracid is easy to synthesize and possesses very good properties [32] such as a high active oxygen content (14.4%) and insusceptibility to shock. The Differential Scanning Calorimeter (DSC) analysis data confirm the non-shock-sensitive nature of nonanebis(peroxoic acid). Details are given in Supporting Information File 1, pages S35–S36. The most important advantage is that it is stable at room temperature. Thus it can be a good alternative for the conventional peracids.

**Scheme 1:**
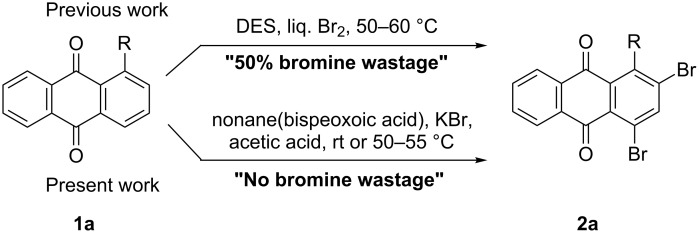
Aliphatic peracid mediated bromination of aminoanthracene-9,10-dinone.

In our previous work [11] (Scheme 1), we reported the use of biodegradable deep eutectic mixtures as an alternative solvent to conventional solvents. The reaction selectively gives dibrominated product along with good recyclability of the solvent. The present work is an extension of our previous work (Scheme 1). Here, we report the use of nonanebis(peroxoic acid) as an oxidant for bromination of aminoanthracene-9,10-dione. To the best of our knowledge, use of this stable, solid peroxy acid and oxidative bromination of amino-anthracene-9,10-dione have not been reported in literature. This oxidation strategy allowed us to brominate various amino anthraquinone derivatives in good yield and at ambient temperature.

## Result and Discussion

The oxidant, nonanebis(peroxoic acid) was synthesized as per the procedure reported in the literature [31]. To confirm the stability of nonanebis(peroxoic acid), one sample was kept in a self-sealing bag at room temperature (30–35 °C). The active oxygen content (% AOC) of the sample was checked by iodometric titration after every 15 days (Table 1). It was found that it retains its active oxygen content over a period of 45 days. There was no change observed in the physical appearance, too. This confirms the stability of nonanebis(peroxoic acid) at room temperature.

**Table 1:** Stability study of nonanebis(peroxoic acid).

Entry	Days	% AOC

1	First day	14.2
2	After 15 days	14.0
3	After 30 days	14.2
4	After 45 days	14.3

A further study was initiated with 1-aminoanthracene-9,10-dione (**1a**) as a substrate and KBr as a source of bromine. Various solvents were screened (Table 2 entry 1–10) among which, acetic acid was found to be more effective than other solvents. It neutralizes the KOH formed during the reaction. To validate the usability of other aliphatic acids, we carried out the reaction in formic, propionic and butyric acid. In the case of formic acid (Table 2, entry 8), during the addition of the peroxy acid, a strong exotherm reaction (temperature rose up to 78–80 °C) was observed. To avoid the high temperature, it is necessary to add the peracid very slowly and cautiously with external cooling. When propionic and butyric acids were used in the reaction, formation of other impurities was observed along with the product **2a**. Considering all these factors, acetic acid was selected as the best solvent for this transformation. The study on peracid molar ratios showed that extra addition of peracid does not influence the yield and reaction time (Table 2, entry 13). When we carried out the reaction at a low temperature (15–18 °C), the rate of reaction remarkably slowed down. HPLC analysis shows 54% conversion after 12 h (Table 2, entry 14). Whereas, at 55 °C, the reaction shows 92% conversion at the expense of 1.25 equivalents of peroxide in 2 h (Table 2, entry 15). No product formation was observed in the absence of peracid (Table 2, entry 16).

**Table 2:** Optimization of reaction conditions.^a^

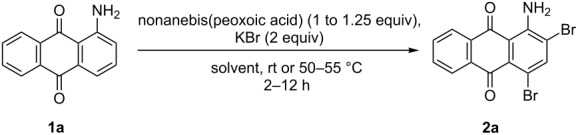						

Entry	Solvent	Oxidant (equiv)	Time (h)	Temp (°C)	Conversion (%)^b^	Yield (%)^c^

1	ACN	1	12/5	rt/50-55	traces	0
2	DMF	1	12/5	rt/50-55	traces	0
3	DCM	1	12/5	rt/40-43	traces	0
4	ethanol	1	12/5	rt/50-55	traces	0
5	glycerol	1	12/5	rt/50-55	nr/traces	0
6	water	1	12/5	rt/50-55	nr	0
7	acetic acid	1	2.30	rt	97	96
8	formic acid	1	2.00	rt	85	90
9	propionic acid	1	2.30	rt	83	85
10	butyric acid	1	2.30	rt	86	87
11	acetic acid	0.5	12	rt	25	45
12	acetic acid	0.75	12	rt	60	68
13	acetic acid	1.25	2.30	rt	95	98
14	acetic acid	1	12	15–18	54	63
15	acetic acid	1.25	2	50–55	92	94
16^d^	acetic acid	–	12/5	rt/50–55	nr	–

^a^Reaction conditions: 1-Aminoanthracene-9,10-dione (2.24 mmol, 1 equiv), KBr (4.48 mmol, 2 equiv), solvent 5 mL; rt: room temperature (30–32 °C); nr: no reaction. ^b^HPLC conversion; ^c^isolated yield. ^d^Reaction carried out in the absence of oxidant. ACN: acetonitrile; DCM: dichloromethane.

To confirm the efficiency of nonanebis(peroxoic acid), bromination was carried out using commercially available oxidants. The results obtained are summarized in Table 3. It was found that one equivalent of nonanebis(peroxoic acid) (contains two active oxygen atoms) (Table 3, entry 1) was sufficient to promote the dibromination of 1-aminoanthracene-9,10-dinone (**1a**) smoothly at room temperature and in less time. Amongst the other oxidants, Oxone (Table 3, entry 2) shows 94% conversion of **1a** in 2 h. In case of 50% Hydrogen peroxide (Table 3, entry 3), more than 7 equivalents of oxidant were required with successive addition. The urea hydrogen peroxide shows moderate conversion in 20 h (Table 3, entry 6). The other diperoxy acids like hexanebis(peroxoic acid) (Table 3, entry 7) and dodecanebis(peroxoic acid) (Table 3, entry 8) have shown poor conversion under present reaction conditions. The bromination of **1a** using performic acid showed traces of conversion while 18% conversion was observed in peracetic acid after 20 h (Table 3, entries 9 and 10). The other oxidants (Table 3, entries 4, 5, 11, 12) showed moderate to poor conversion.

**Table 3:** Comparison with commercially available oxidants.^a^

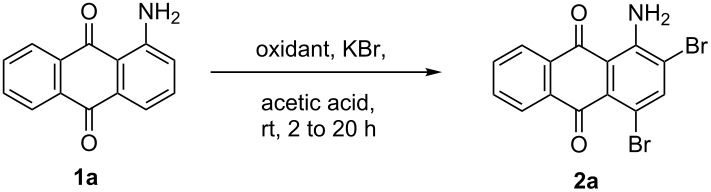				

Entry	Oxidant	Oxidant (equiv)	Time (h)	Conversion (%)^b^

1	nonanebis peroxoic acid	1	2.3	97
2	Oxone	2	2	94
3	50% H_2_O_2_	>7	20	86
4	sodium perborate	2	20	20
5	ammonium persulfate	2	20	3
6	urea hydrogen peroxide	2	20	85
7	hexanebis(peroxoic acid)	1	20	20
8	dodecanebis(peroxoic acid)	1	20	58
9	performic acid	2	20	traces
10	peracetic acid	2	20	18
11	*m*-CPBA	2	20	43
12	70% TBHP	2	20	15

^a^Reaction conditions: 1-Aminoanthracene-9,10-dinone (0.5 g, 2.24 mmol), KBr (0.53 g, 4.48 mmol), oxidant (1 to 7 equiv), acetic acid 5 mL; temperature: room temperature (30–32 °C); reaction monitored on TLC. ^b^HPLC conversion.

The optimized conditions have a broad substrate scope which was investigated by examining various amino substituted derivatives of anthracene-9,10-dione. In most cases, under standard conditions, the reaction proceeded smoothly at room temperature in few hours. The unsubstituted amine derivatives **1a–1f** (Table 4, entries 1–6) and amine with an electron donating substituent at the *para*-position (**1g**, Table 4, entry 7) were brominated at room temperature and gave an excellent yield of the desired product. Whereas, the amine with an electron withdrawing substituent at *para*-position (**1h**, Table 4, entry 8) or N-alkylated amines **1i** and **1j** (Table 4, entries 9 and 10) showed very low conversion at room temperature even after carrying out the reaction for 12 h. The rate of bromination of these derivatives (**1h–1j**) can be increased by carrying out bromination at 50–55 °C. The electron deficient arene **1k** (Table 4, entry 11) also undergoes bromination at 50–55 °C. In case of substrates like 1,4-diaminoanthracene-9,10-dione and 1-amino-4-hydroxyanthracene-9,10-dione, the protocol was found to be unsuccessful. It was surmised that in the presence of oxidant these substrates form a diimine type product (similar to oxidative hair dye mechanism) [33], which makes the ring unreactive towards electrophilic substitution.

Since Oxone and 50% hydrogen peroxide showed good results with substrate **1a**, we have checked the applicability of these two oxidants for the bromination of some substrates listed in Table 4. The bromination of substrate **1b** using Oxone as an oxidant required 14 h for complete conversion at room temperature. Whereas, the substrate **1g** did not show complete conversion in 24 h at room temperature as well as at 50–55 °C. The N-alkylated substrates such as **1i**, **1j** and the substrate **1k** showed poor conversion (product formed in tracess) even at a higher temperature when 50% hydrogen peroxide was used as an oxidant. This study demonstrates the superiority of nonanebis(peroxoic acid) under the present protocol.

**Table 4:** Bromination of various aminoanthracene-9,10-dione derivatives and benzanthrone.^a^

Entry	Reactant	Product	Time/Temp (h/°C)	Yield (%)^b^	HPLC (%)	mp (°C)	
						Obs. (°C)	Rep. (°C)

1	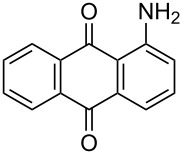 **1a**	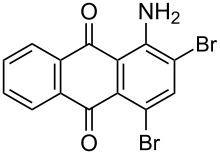 **2a**	2.30/rt	92	97	223–224	224–226 [11]
2	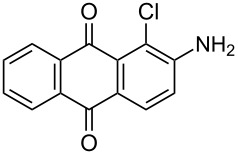 **1b**	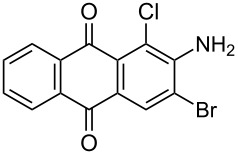 **2b**	2.00/rt	97	99	235–237	238 [17]
3	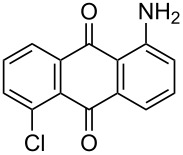 **1c**	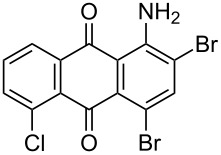 **2c**	3.00/rt	93	98	246–248	249–250 [11]
4	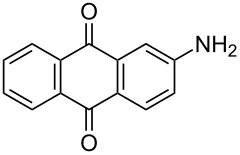 **1d**	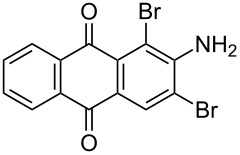 **2d**	2.00/rt	96	99	250	250 [34]
5	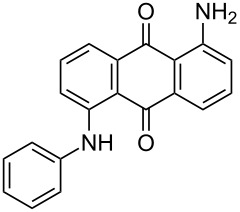 **1e**	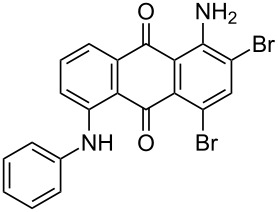 **2e**	2.00/rt	87	98	209–210	–
6	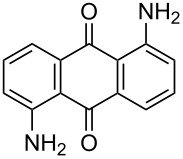 **1f**	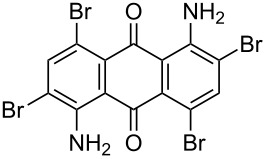 **2f**	3.00/rt	80	99	263–265	Decomp. >250 [11]
7	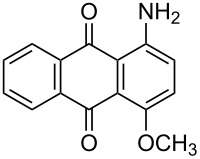 **1g**	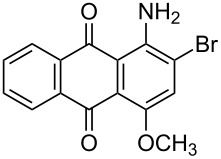 **2g**	3.00/rt	86	99	177–178	174–176 [11]
8	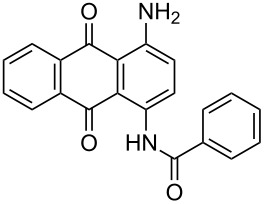 **1h**	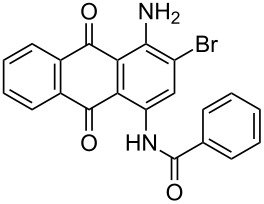 **2h**	3.00/50–55	83	99	246–248	247 [11]
9	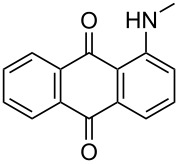 **1i**	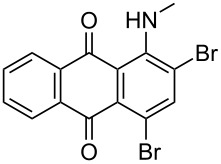 **2i**	2.30/50–55	86	98	153–154	154–158 [11]
10	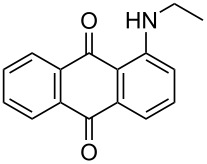 **1j**	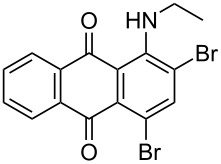 **2j**	2.30/50–55 °C	93	99	136–137	–
11	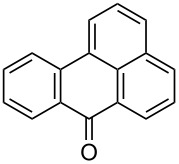 **1k**	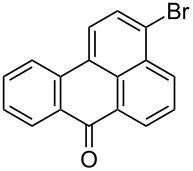 **2k**	3.00/50–55 °C	86	99	177	177 [35]

^a^Reaction conditions: Aminoanthracene-9,10-dione (0.5 g, 1 equiv); acetic acid (5 mL); KBr [1 equiv (for entries 2, 7, 8, 11); 2 equiv (for entries 1, 3, 4, 5, 9, 10); 4 equiv (for entry 6)]; nonanebis(peroxoic acid) [0.5 equiv (for entries 2, 7); 0.8 equiv (for entries 8, 11); 1 equiv (for entries 1, 3, 4, 5); 1.2 equiv (for entries 9,10); 2 equiv (for entry 6)]; rt: room temperature (30–32 °C); ^b^isolated yield.

**Scheme 2:**
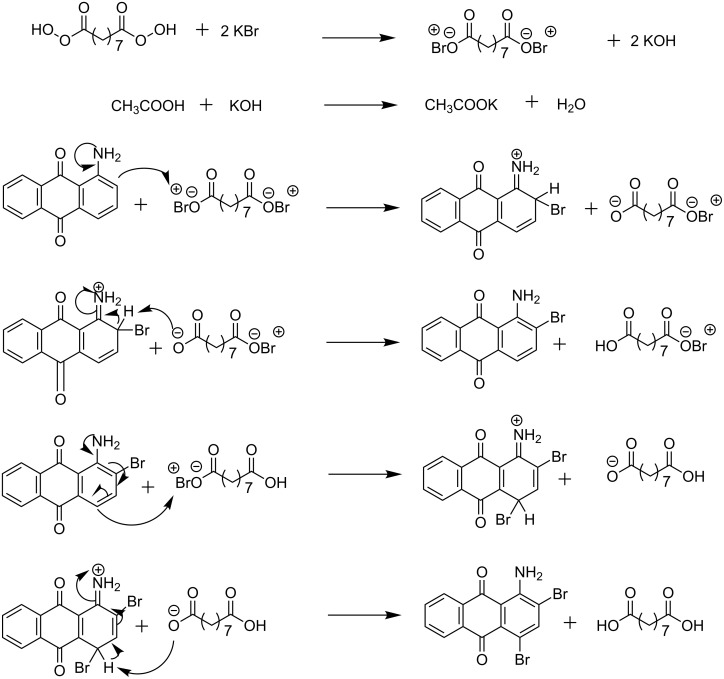
Plausible mechanism for the bromination of aminoanthracene-9,10-dione [36–37].

It was surmised that, the oxidant nonanebis(peroxoic acid) oxidizes the KBr to Br^+^ (Scheme 2). During this process, KOH is formed, which is neutralized by the acetic acid. The bromonium ion thus formed reacts with the amine to give the brominated product. In case of unsubstituted amines, *ortho-*bromination occurs first, while in case of N-substituted amines *para-*bromination takes place first [22].

## Conclusion

Thus, we can summarize that we have developed a simple, efficient and convenient process for the bromination of aminoanthracene-9,10-dione using nonanebis(peroxoic acid) as an oxidant. The bromination reaction proceeds under mild conditions with high yield and purity. The peracid used here is stable, safe and easy to handle and thus can be a good attractive alternative to the conventional peracids. Further study on nonanebis(peroxoic acid) is under progress.

## Experimental

### General information

All products were confirmed by melting point, FTIR spectroscopy, ^1^H NMR spectroscopy and mass spectrometry. Purity was checked by HPLC using acetonitrile/methanol (90:10) mobile phase and silica column C18. All melting points are uncorrected and are presented in degree Celsius. ^1^H NMR (400 MHz) spectrum were recorded using Varian spectraprobe and Bruker 400 ultrashield (Advance 400 NMR spectrometer) and chemical shifts are expressed in δ ppm using TMS as an internal standard. Mass spectral data were obtained with a Micromass Q-TOF spectrometer. The DSC analysis was done using a DSC Q100 V9.9 Build 303 (Universal V4.5A TA Instrument), ramp 10.00 °C/min to 300.00 °C, flow rate: 50.0 mL/min.

**Procedure for the preparation of nonanebis(peroxoic acid):** In 250 mL round bottomed flask equipped with a mercury sealed stirrer, 10 g of nonanedioic acid (0.0531 mol) was dissolved in 95% sulfuric acid (25 g, 0.255 mol) with good stirring. The reaction mass was cooled to 15 °C using an ice-water bath. To this 65% hydrogen peroxide (11 g, 0.323 mol) was added drop-wise while maintaining the internal temperature at 15 to 20 °C. After the addition, the reaction mass was further stirred for 5 h at 15 to 20 °C. Then, 50 mL half saturated aqueous ammonium sulfate solution (35 g/100 g water) was added to the reaction mass at 0 °C. The white solid was filtered and washed with cold half saturated ammonium sulfate solution (4 × 10 mL). The crude product obtained was dried under vacuum at room temperature to give the final product (9.2 g, 78% yield).

The active oxygen content of the final product was determined by iodometric titration. (Reported: 14.5%; Obtained: 14.2–14.4%.)

**General procedure for oxidative bromination of aminoanthracene-9,10-dione:** In a 100 mL round bottomed flask equipped with a mercury sealed stirrer, 5 mL acetic acid was placed to which aminoanthracene-9,10-dione (**1**, 1 equiv), and KBr (0.5 to 2 equiv) were added under stirring. To this slurry, nonanebis(peroxoic acid) (1 to 2 equiv) was added cautiously over a period of 10 min at room temperature. The reaction mass was stirred at room temperature/50–55 °C and was monitored by TLC. After completion, the reaction mass was poured into 10% sodium bicarbonate solution to neutralize the acids. The solid obtained was filtered and washed with water till neutral pH was obtained. The crude product was purified by column chromatography on a silica gel with hexane/ethyl acetate (95:5) as an eluent to give purified product **2**.

### Scale-up study

The reaction of 1-aminoanthracene-9,10-dione with KBr in acetic acid and nonanebis(peroxoic acid) as an oxidant was scaled-up to 10 g to understand the functioning of the method on a larger scale. The reaction yields 96% desired dibromo product without formation of any isomer in 2.30 h at room temperature. From this it was clear that the present protocol shows good applicability on larger scale also.

## Supporting Information

File 1 Characterization details for all the products in Table 4.

File 2 FTIR, mass and ^1^H NMR spectra of all products in Table 4, Differential Scanning Colorimetric (DSC) analysis and calculations for shock sensitivity of nonanebis(peroxoic acid), HPLC analysis details, procedure for the detection of % active oxygen content (%AOC).
