# Supplementary material for: Nonanebis(peroxoic acid): a stable peracid for oxidative bromination of aminoanthracene-9,10-dione
Source: Beilstein J Org Chem. 2014 Apr 24;10:921–8. doi: 10.3762/bjoc.10.90 (PMC4077418; doi:10.3762/bjoc.10.90)
Supplement: File 1 — Characterization details for all the products in Table 4. [file Beilstein_J_Org_Chem-10-921-s001.pdf]

Supporting Information File 1

for

Nonanebis(peroxoic acid): a stable peracid for oxidative bromination of aminoanthracene-9,10-dione

Vilas Venunath Patil and Ganapati Subray Shankarling*

Address: Department of Dyestuff Technology, Institute of Chemical Technology, N. P. Marg, Matunga, Mumbai - 400019, India. Tel.: 91-22-33612708, Fax: 91-22-33611020

E-mail: Ganapati S. Shankarling* - gsshankarling@gmail.com

*Corresponding author

Characterization details for all the products in Table 4

Experimental details:

1-Amino-2,4-dibromoanthracene-9,10-dione (2a). As per general procedure, the reaction of **1a** (2.24 mmol, 1 equiv) with KBr (4.48 mmol, 2 equiv) and nonanebis(peroxoic acid) (2.24 mmol, 1 equiv) in 5 mL acetic acid at 30–32 °C gives orange colored solid **2a** (0.79 g, 92% yield): mp (measured) 224 °C (from hexane); IR **:** λmax/cm^{-1} 3395 (NH), 1670.05 and 1580.38 (CO), 715.4 (C-Br); ^{1}H NMR : (400MHz, DMSO) δ(ppm): 7.88-7.91 (d, 2H, Ar-H), 8.10-8.19 (t, 2H, Ar-H); 8.23 (s, 1H, Ar-H); MS : m/z (EI) 380.1 (M + H) for ^{79}Br. $C_{14}H_7Br_2NO_2$ calculated m/z: 379.2.

2-Amino-3-bromo-1-chloroanthracene-9,10-dione (2b). As per general procedure, the reaction of **1b** (1.94 mmol, 1 equiv) with KBr (1.94 mmol, 1 equiv) and nonanebis(peroxoic acid) (0.97 mmol, 0.5 equiv) in 5 mL acetic acid at 30–32 °C gives yellow colored solid **2b** (0.60 g, 92% yield) : mp (measured) 238 °C (from hexane); IR **:** λmax/cm^{-1} 3489 and 3384 (NH), 1671 and 1653 (CO), 789.7 (C-Br), 814.77 (C-Cl) ; ^{1}H-NMR : (400MHz, DMSO) δ(ppm): 6.86 (s, 2H, weak, -N-H), 7.87 – 7.89 (d, 2H, Ar-H), 8.10-8.14 (t, 2H, Ar-H), 8.22 (s, 1H, Ar-H)**;** MS: m/z (EI) 336.1 (M + H) for ^{79}Br, ^{35}Cl. $C_{14}H_7BrClNO_2$ calculated m/z: 334.9.

1-Amino-2, 4-dibromo-5-chloroanthracene-9,10-dione (2c). As per general procedure, the reaction of **1c** (1.94 mmol, 1 equiv.) with KBr (3.88 mmol, 2 equiv.) and nonanebis(peroxoic acid)(1.94 mmol, 1 equiv.) in 5 ml acetic acid at 30 to 32 °C gives orange colored solid **2c** (0.75g, 93% yield) : mp (measured) 246 °C (From Hexane); IR: λmax/cm^{-1} 3450 and 3304 (NH), 1675 and 1639 (CO), 739 (C-Br), 810.9(C- Cl); ^{1}H NMR : (400MHz, $CDCl_3$) δ(ppm): 7.6-7.65 (t, 1H), 7.7 – 7.8 (t, 1H, Ar-H), 8.05 (s, 1H, Ar-H), 8.2 (d, 1H, Ar-H); MS : m/z (EI) 414.1 (M + H) for ^{79}Br, ^{35}Cl. $C_{14}H_6Br_2ClNO_2$ calculated m/z: 412.8.

2-Amino-1,3-dibromoanthracene-9,10-dione (2d). As per general procedure, the reaction of **1d** (2.24 mmol, 1 equiv) with KBr (4.48 mmol, 2 equiv) and nonanebis(peroxoic acid) (3.36 mmol, 1.5 equiv) in 5 mL acetic acid at 30– 32 °C gives orange yellow colored solid **2d** (0.79g, 96% yield) : mp (measured) 250 °C (from hexane); IR : λmax/cm-1 3479, 3377 (NH), 1672 and 1581 (CO), 705 (C-Br); ^{1}H NMR : (400MHz, DMSO) δ(ppm): 6.755 (s, 2H, N-H), 7.86 - 7.89 (m, 2H, Ar-H), 8.09 – 8.14 (m, 2H, Ar-H), 8.26 (s, 1H, Ar-H) ; MS : m/z (EI) 380.1 (M + H) for ^{79}Br. $C_{14}H_7Br_2NO_2$ calculated m/z: 379.2.

1-Amino-2,4-dibromo-5-(phenylamino)anthracene-9,10-dione (2e): As per general procedure, the reaction of **1e** (1.59 mmol, 1 equiv) with KBr (3.18 mmol, 2 equiv) and nonanebis(peroxoic acid) (1.91 mmol, 1.1 equiv.) in 5 mL acetic acid at 30– 32 °C gives brown colored solid **2e**(0.94 g , 80% yield) : mp 209-210 °C (from hexane). IR : λmax/cm-1 3477 and 3319 (NH), 715 (C-Br); 1H-NMR: (400MHz, CDCl3) δ (ppm): 7.26-7.60 (m, 4H, Ar-H), 7.78-7.84 (m, 2H, Ar-H), 8.085 (s, 1H, Ar-H), 8.10-8.15 (d, 2H, Ar-H), 9.25-9.32 (dd, 1H, Ar-H) 12.83 (s, weak, N-H), 13.26 (s, weak, N-H); MS: m/z (EI) 469.2 (M - H) for 79Br. $C_{20}H_{12}Br_2N_2O_2$ calculated m/z: 469.9.

1,5-Diamino-2,4,6,8-tetrabromoanthracene-9,10-dione (2f): As per general procedure, the reaction of **1f** (2.1 mmol, 1 equiv) with KBr (8.4 mmol, 4 equiv) and nonanebis(peroxoic acid) (4.2 mmol, 2 equiv) in 5 mL acetic acid at 30– 32 °C gives brown colored solid **2f** (0.94 g, 80% yield): mp (measured on DSC) 263.5 °C (from hexane) (lit. 249.5 °C); IR : λmax/cm^{-1} 3477 and 3319 (NH), 715 (C-Br); 3477 and 3319 (NH), 1670 and 1580 (CO), 715 (C-Br); ^{1}H NMR : (400MHz, DMSO) δ(ppm): 7.36–7.97 (2H, m, Ar-H); MS : m/z (EI) 551.5 (M + H) for ^{79}Br. $C_{14}H_6Br_4N_2O_2$ calculated m/z: 550.2.

1-Amino-2-bromo-4-methoxyanthracene-9,10-dione (2g): As per general procedure, the reaction of **1g** (1.97 mmol, 1 equiv) with KBr (1.48 mmol, 0.75 equiv) and nonanebis(peroxoic acid) (1.58 mmol, 0.8 equiv) in 5 ml acetic acid at 30–32 °C gives brown colored solid **2g** (0.57 g, 86% yield) : mp (measured) 178 °C (from hexane); IR : λmax/cm^{-1} 3435 and 3285 (NH), 1659 and 1625 (CO), 782 (C-Br); ^{1}H NMR : (400MHz, DMSO) δ(ppm): 3.98 (s, 3H, -OCH_3), 4.06 (s, 1H, N-H), 7.62 (s, 1H, Ar-H), 7.73-7.74 (d, 2H, Ar-H), 8.22-8.26 (m, 2H, Ar-H); MS: m/z (EI) 332.2 (M + H) for ^{79}Br. $C_{15}H_{10}BrNO_3$ calculated m/z: 330.9.

***N*-(4-Amino-3-bromo-9,10-dioxo-9,10-dihydroanthracen-1-yl)benzamide (2h).** As per general procedure, the reaction of **1h** (1.46 mmol, 1 equiv) with KBr (1.24 mmol, 0.85 equiv) and nonanebis(peroxoic acid) (1.6 mmol, 1.1 equiv) in 5 mL acetic acid at 50–55 °C gives brown colored solid **2h** (0.51 g, 83% yield) : mp (measured) 248 °C (from toluene); IR : λmax/cm^{-1} 3464.49 and 3269.72 (NH), 1669.09.05 and 1628.59 (CO), 735.71 (C-Br); ^{1}H NMR : (400MHz, DMSO) δ(ppm): 7.64- 7.67 (m, 3H, Ar-H), 7.87-7.93 (d, 2H, Ar-H), 8.02-8.05 (d, 2H, Ar-H), 8.21-8.23 (t, 2H, Ar-H), 9.41 (s, 1H, Ar-H), 13.1 (s, 1H, -CONH); MS: m/z (EI) 421.3 (M + H) for ^{79}Br. $C_{21}H_{13}BrN_2O_3$ calculated m/z: 420.01.

2,4-Dibromo-1-(methylamino)anthracene-9,10-dione (2i). As per general procedure, the reaction of **1i** (2.1 mmol, 1 equiv) with KBr (4.21 mmol, 2 equiv) and nonanebis(peroxoic acid) (2.52 mmol, 1.2 equiv) in 5 mL acetic acid at 50–55 °C gives brown colored solid **2i** (0.724 g, 87% yield) : mp (measured) 154 °C (from hexane); IR : λmax/cm^{-1}3456 (NH), 1672

and 1625 (CO), 737 (C-Br); ^{1}H NMR : (400Mz, $CDCl_3$) δ(ppm): 3.06 (s, 3H, $-CH_3$), 7.85-7.88 (d, 2H, Ar-H), 8.09-8.12 (t, 2H, Ar-H), 8.16 (s, 1H, Ar-H), 8.98 (s, 1H, -NH); MS : m/z (EI) 394.1 (M + H) for ^{79}Br. $C_{15}H_9Br_2NO_2$ calculated m/z: 393.2

2,4-Dibromo-1-(ethylamino)anthracene-9,10-dione (2j). As per general procedure, the reaction of **1j** (1.99 mmol, 1 equiv) with KBr (3.97 mmol, 2 equiv) and nonanebis(peroxoic acid) (2.38 mmol, 1.2 equiv) in 5 mL acetic acid at 50–55 °C gives brown colored solid **2j** (0.76 g , 93% yield) : mp (measured) 158 °C (from hexane); IR : λmax/cm^{-1} 3460 (NH), 1671 and 1626 (CO), 738 (C-Br); ^{1}H NMR : (400MHz, DMSO) δ(ppm): 1.3-1.4 (t, 3H, $-CH_3$), 3.7-3.8 (q, 2H, $-CH_2$) 7.7-7.8 (d, 2H, Ar-H), 8.1-8.18 (s, 1H, Ar-H), 8.2-8.3 (t, 2h, Ar-H), 9.9 (s, 1H, -NH); MS : m/z (EI) 408.1 (M + H) for ^{79}Br. $C_{16}H_{11}Br_2NO_2$ calculated m/z: 406.9.

3-Bromo-7*H*-benzo[*d,e*]anthracen-7-one (2k): As per general procedure, the reaction of **1k** (2.17 mmol, 1 equiv.) with KBr (2.38 mmol, 1.1 equiv) and nonanebis(peroxoic acid) (2.6 mmol, 1.2 equiv) in 5 mL acetic acid at 50–55 °C gives dark yellow colored solid **2k** (0.61 g, 88% yield) : mp (measured) 168 °C (from hexane); IR : λmax/cm^{-1} 1649.8 (CO), 780 (C-Br); ^{1}H NMR : (400MHz, DMSO) δ(ppm): 7.64-7.66 (t, 1H, Ar-H), 7.85-7.90 (t, 1H, Ar-H), 8.01-8.06 (t, 1H, Ar-H), 8.15-8.18 (d, 1H, Ar-H), 8.31-8.34 (d, 1H, Ar-H), 8.6-8.72 (m, 4H, Ar-H); MS : m/z (EI) 309.2 (M + H) for ^{79}Br. $C_{17}H_9BrO$ calculated m/z: 307.9.
