# Supplementary material for: Nonanebis(peroxoic acid): a stable peracid for oxidative bromination of aminoanthracene-9,10-dione
Source: Beilstein J Org Chem. 2014 Apr 24;10:921–8. doi: 10.3762/bjoc.10.90 (PMC4077418; doi:10.3762/bjoc.10.90)
Supplement: File 2 — FTIR, mass and 1H NMR spectra of all products in Table 4, Differential Scanning Colorimetric (DSC) analysis and calculations for shock sensitivity of nonanebis(peroxoic acid), HPLC analysis details, procedure for the detection of % active oxygen content (%AOC). [file Beilstein_J_Org_Chem-10-921-s002.pdf]

Supporting Information File 2

for

Nonanebis(peroxoic acid): a stable peracid for oxidative bromination of aminoanthracene-9,10-dione

Vilas Venunath Patil and Ganapati Subray Shankarling*

Address: Department of Dyestuff Technology, Institute of Chemical Technology, N. P. Marg, Matunga, Mumbai - 400019, India. Tel.: 91-22-33612708, Fax: 91-22-33611020

E-mail: Ganapati S. Shankarling* - gsshankarling@gmail.com

*Corresponding author

FTIR, mass and ^{1}H NMR spectra of all products in Table 4, Differential Scanning Colorimetric (DSC) analysis and calculations for shock sensitivity of nonanebis(peroxoic acid), HPLC analysis details, procedure for the detection of % active oxygen content (%AOC).

Table of Contents

FTIR Spectra	S2–S12
EIMS Spectra	S13–S23
^{1}H NMR Spectra	S24–S34
DSC analysis of nonanebis(peroxoic acid)	S35–S36
HPLC Analysis specification	S37
Determination of % Active Oxygen Content	S38
Reference	S39

FTIR Spectra

1) 1-Amino-2,4-dibromoanthracene-9,10-dione (2a)

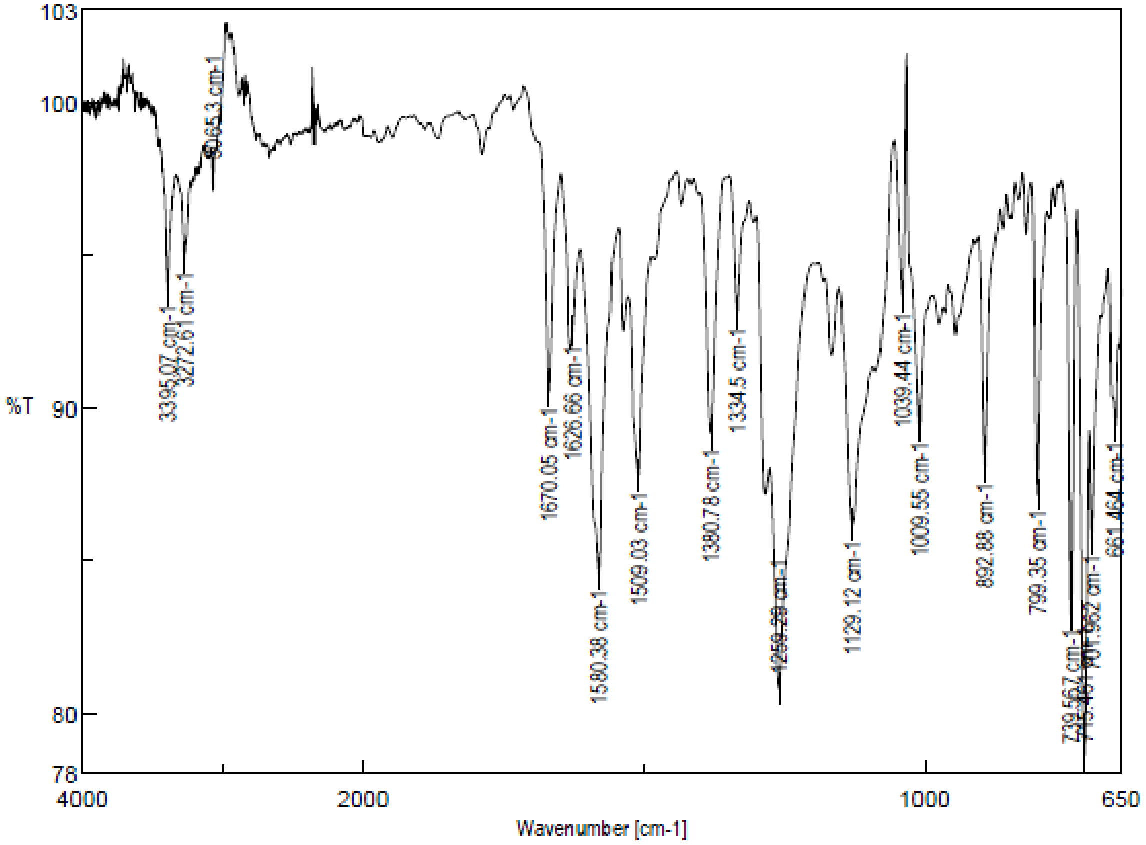

2) 2-Amino-3-bromo-1-chloroanthracene-9,10-dione (2b)

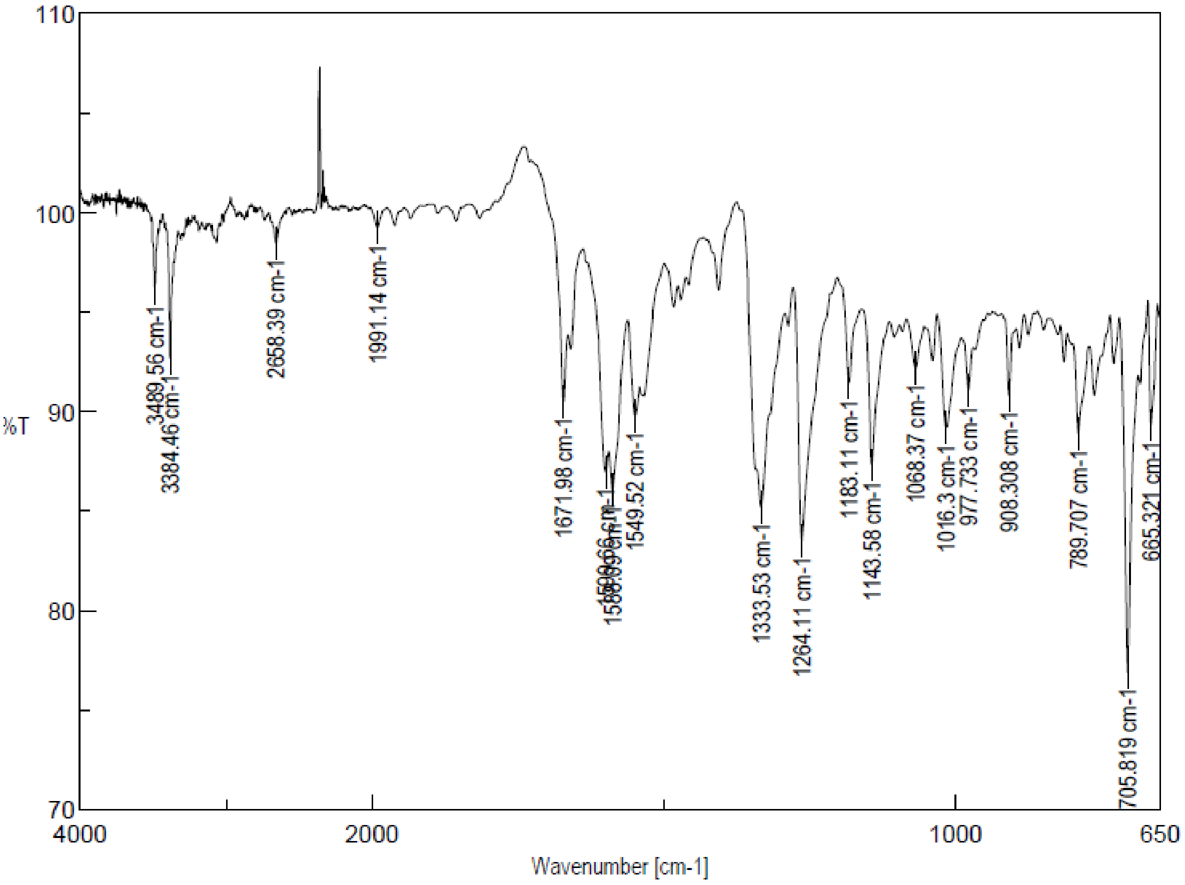

3) 1-Amino-2, 4-dibromo-5-chloroanthracene-9,10-dione (2c)

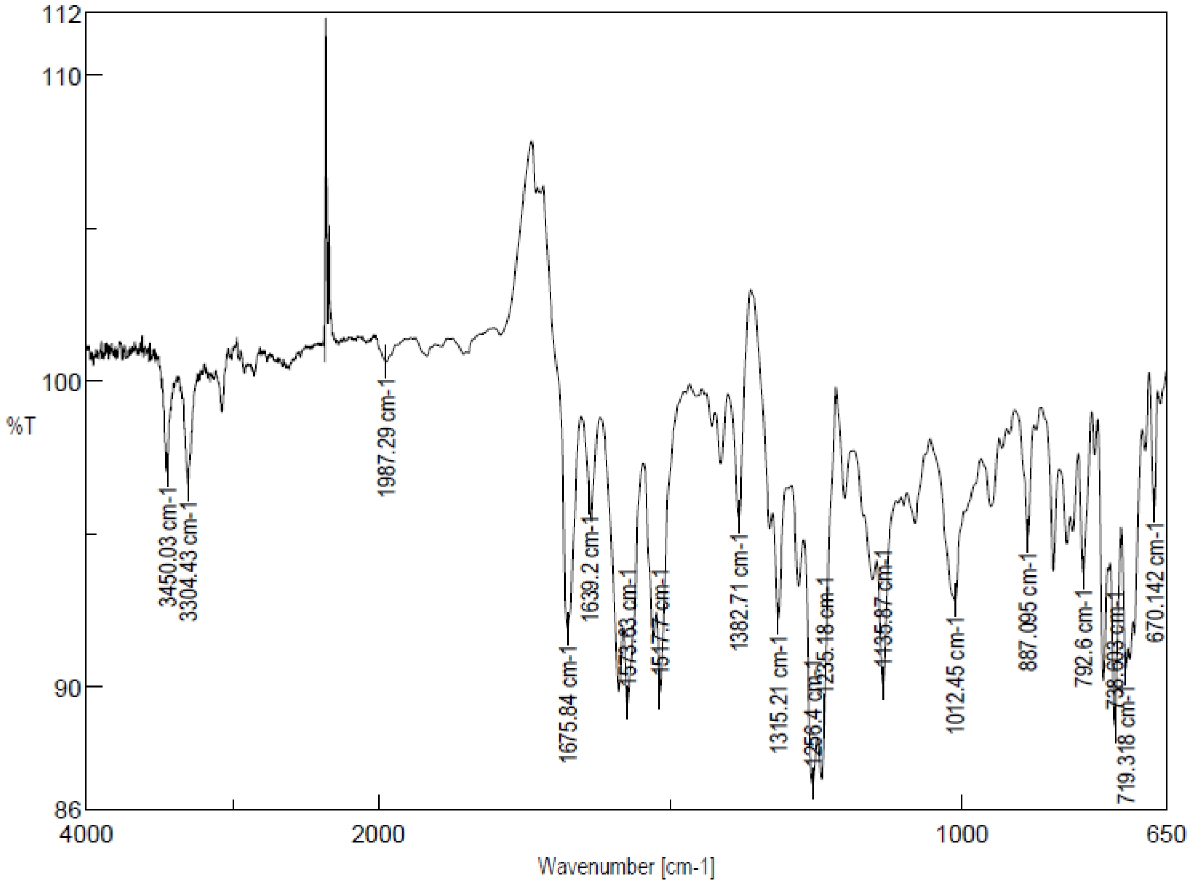

4) 2-Amino-1,3-dibromoanthracene-9,10-dione (2d)

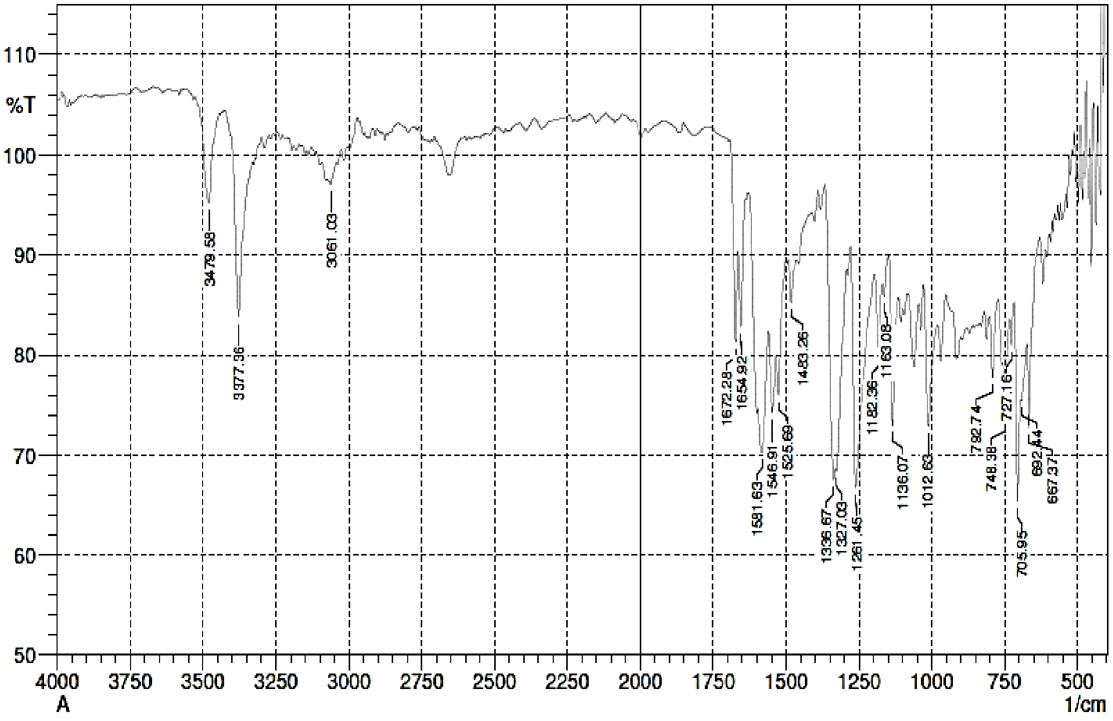

5) **1-Amino-2,4-dibromo-5-(phenylamino)anthracene-9,10-dione (2e)**

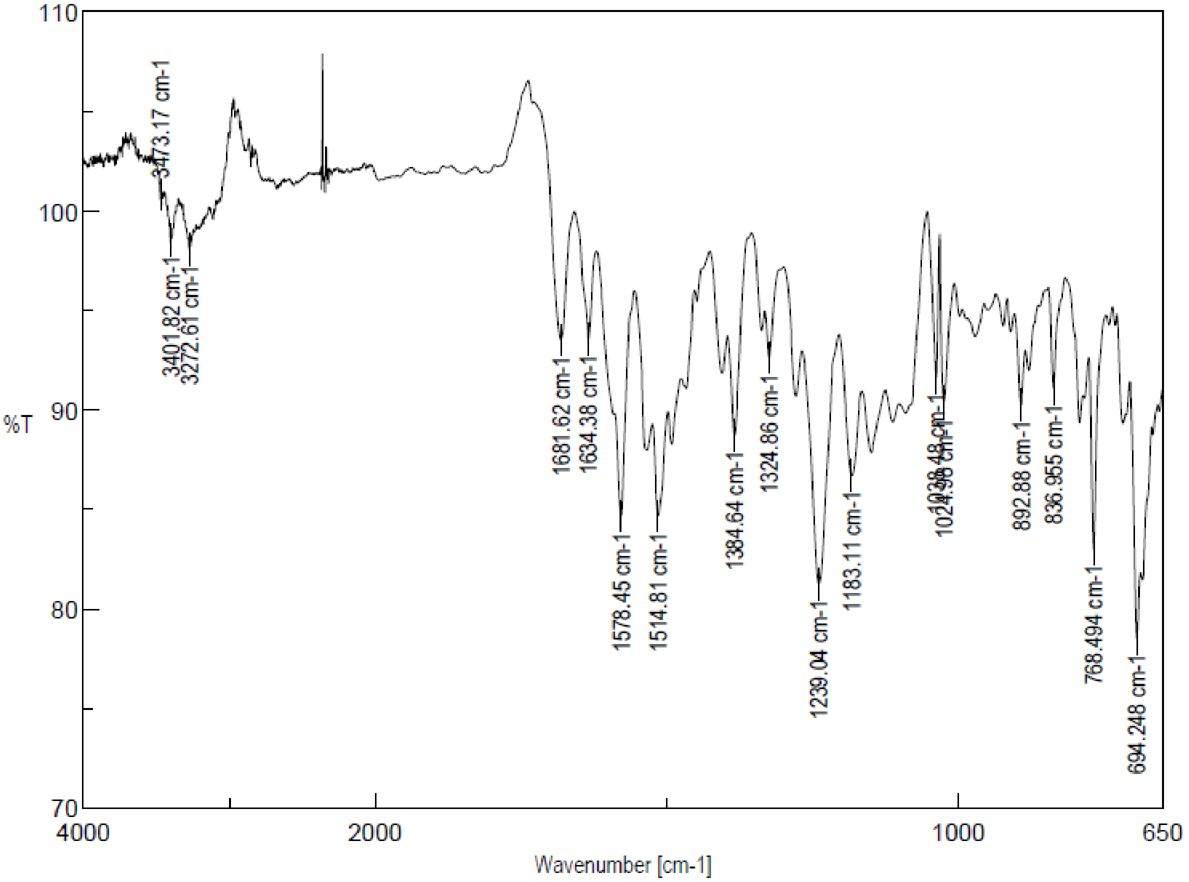

6) 1,5-Diamino-2,4,6,8-tetrabromoanthracene-9,10-dione (2f)

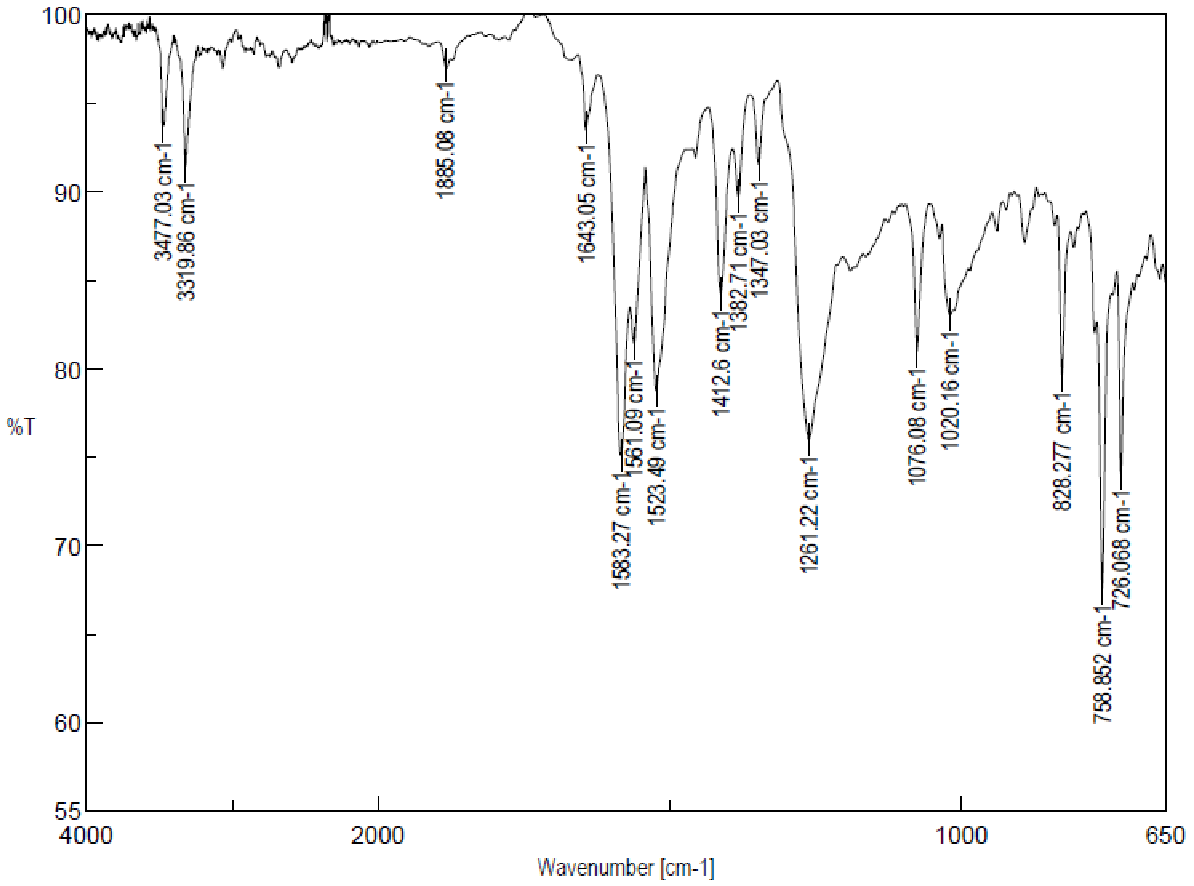

7) 1-Amino-2-bromo-4-methoxyanthracene-9,10-dione (2g)

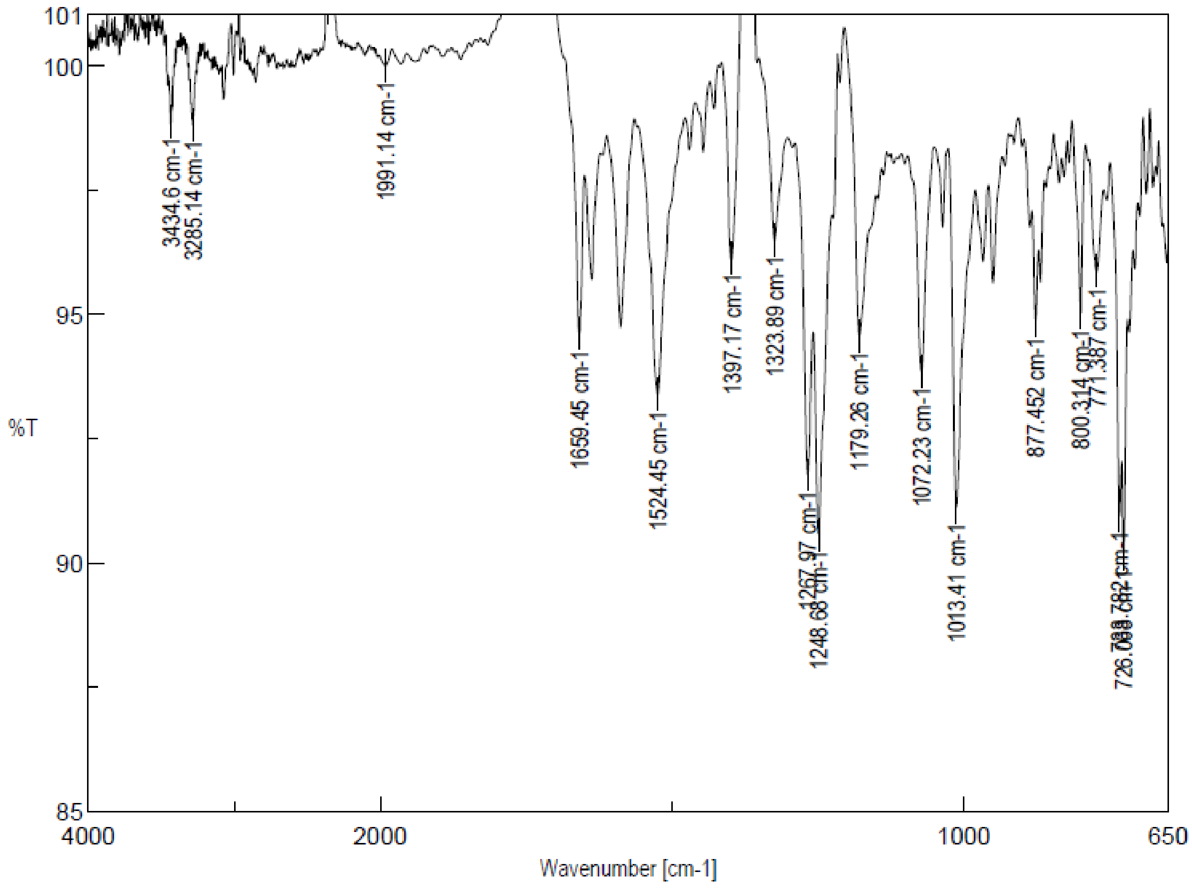

1-

8) *N*-(4-amino-3-bromo-9,10-dioxo-9,10-dihydroanthracen-1-yl)benzamide (2h)

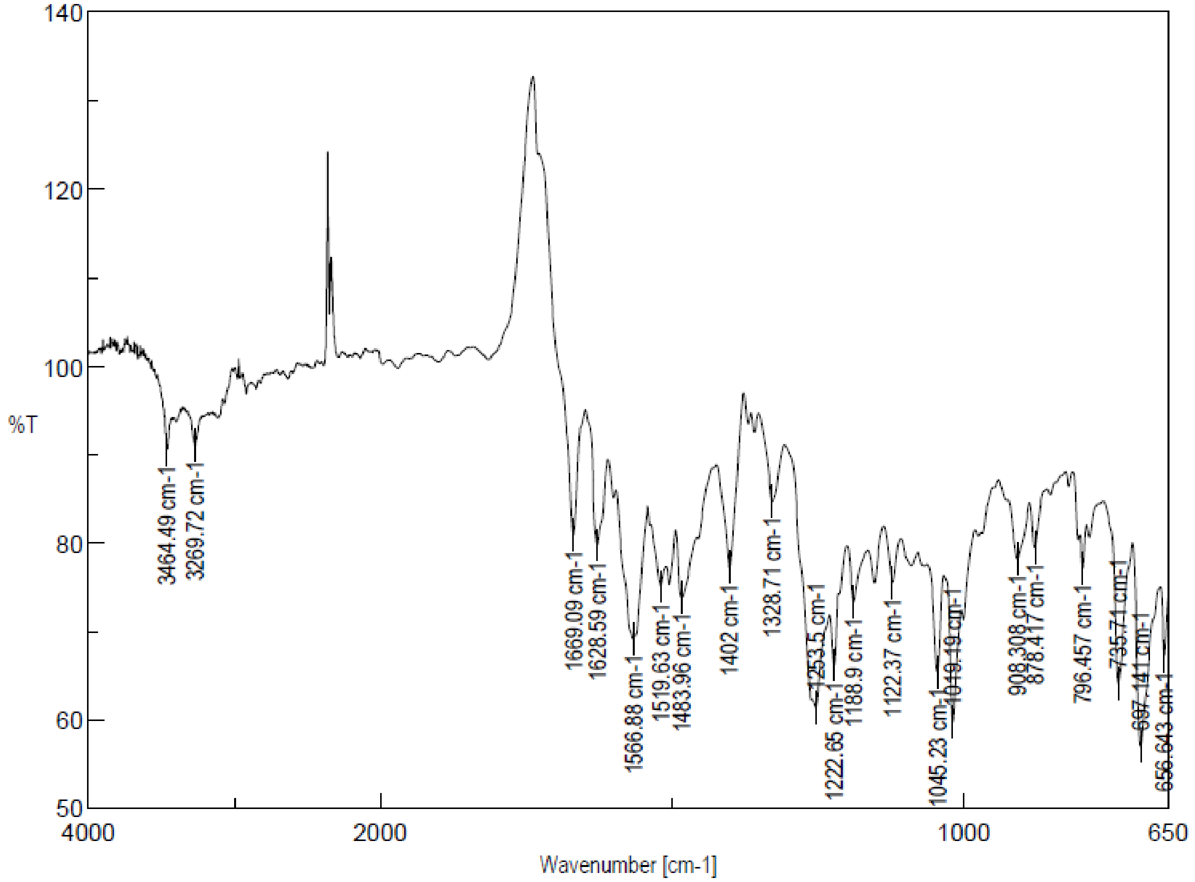

9) 2,4-Dibromo-1-(methylamino)anthracene-9,10-dione (2i)

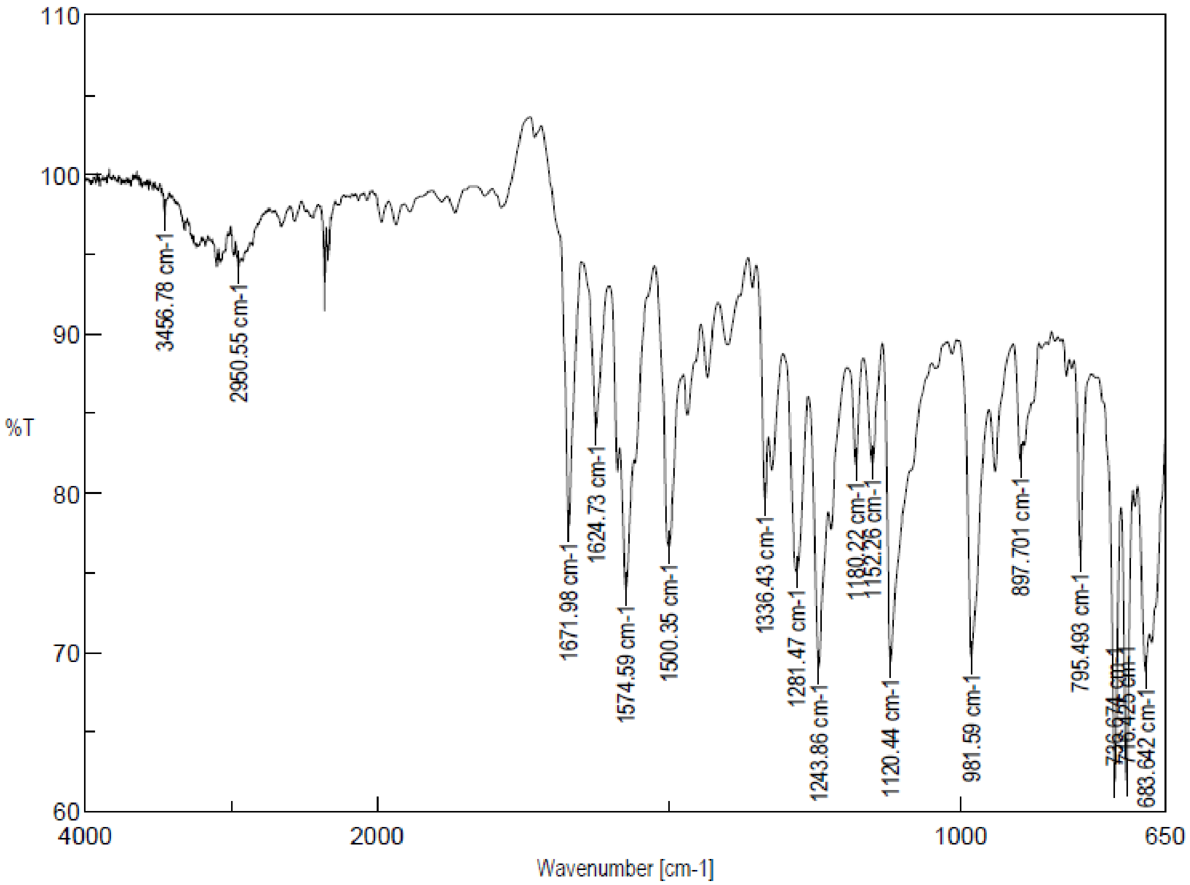

10) 2,4-Dibromo-1-(ethylamino)anthracene-9,10-dione (2j)

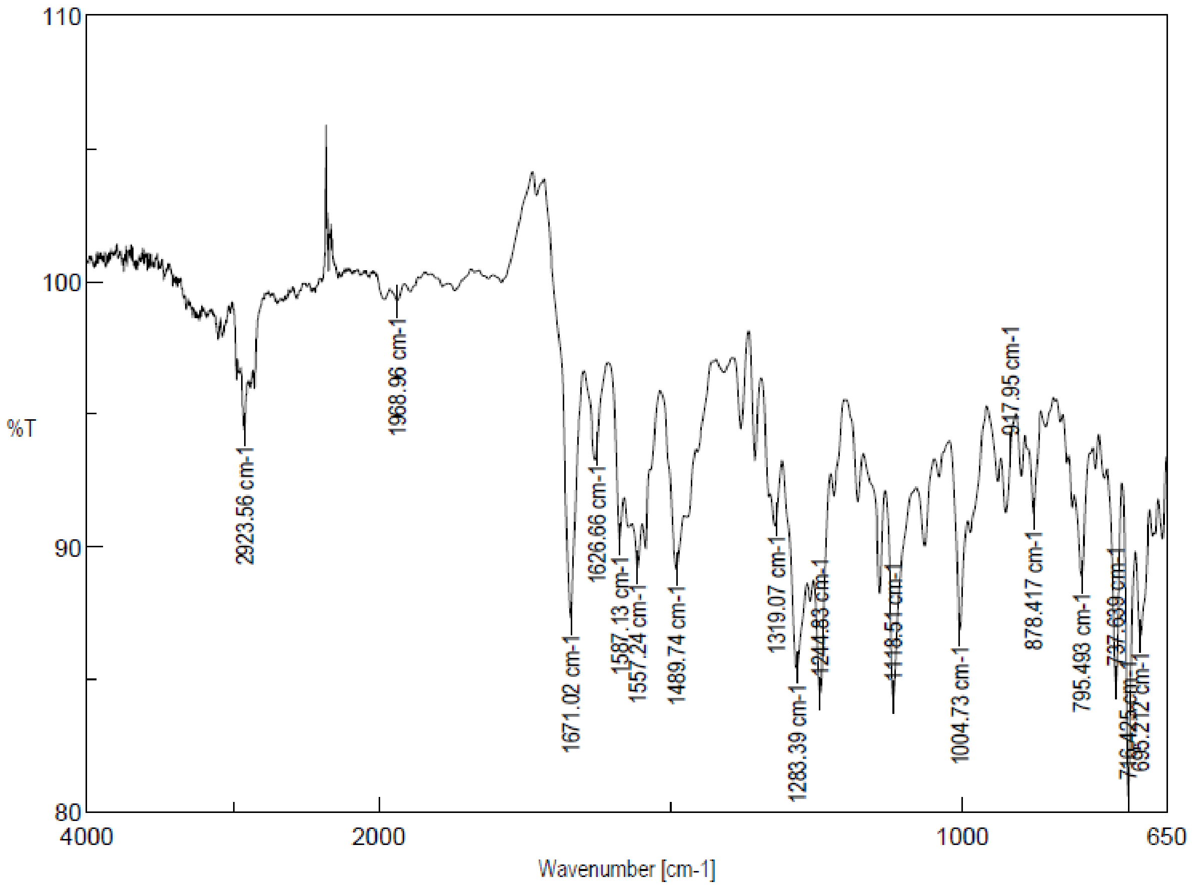

11) 3-Bromo-7*H*-benzo[*d*,*e*]anthracen-7-one (2k)

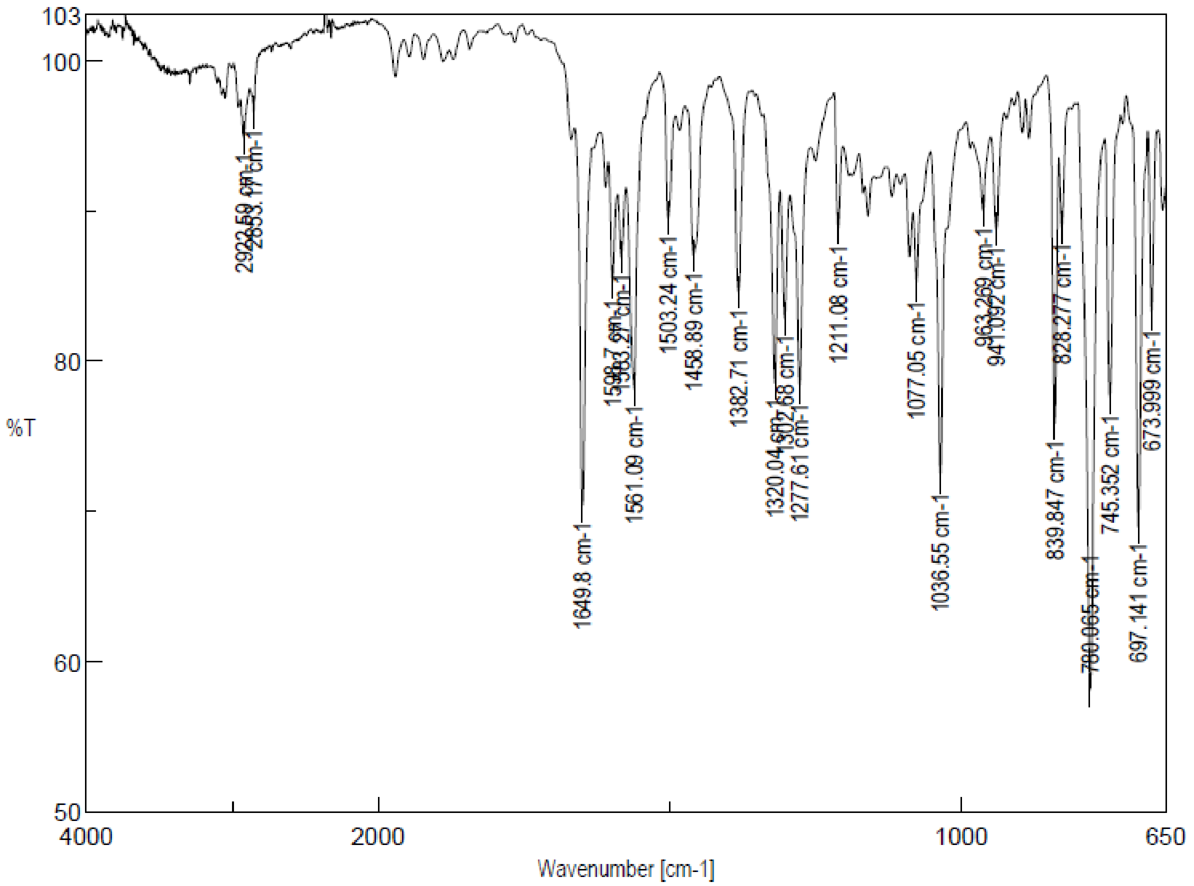

EIMS Spectra

1) **1-Amino-2,4-dibromoanthracene-9,10-dione (2a)**

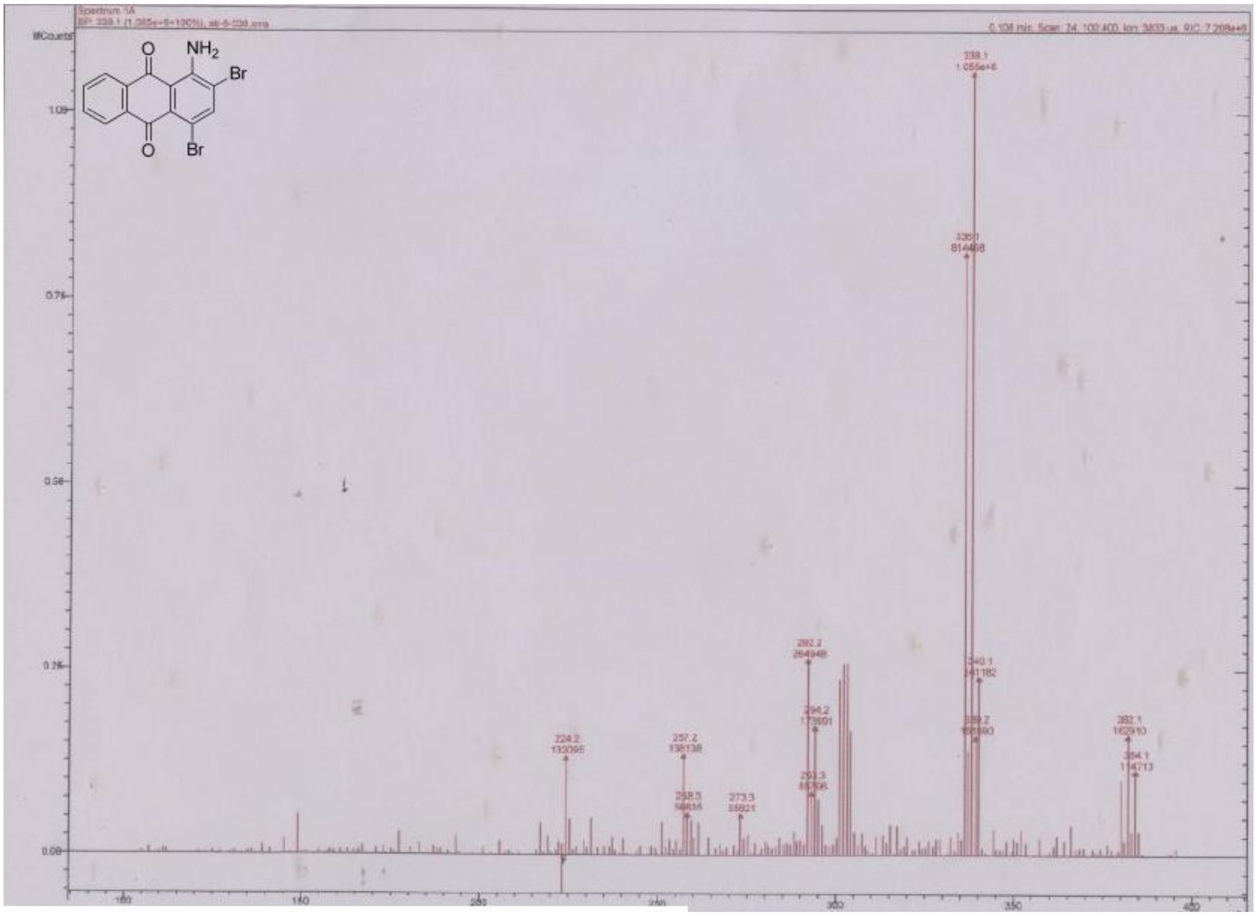

2) 2-Amino-3-bromo-1-chloroanthracene-9,10-dione (2b)

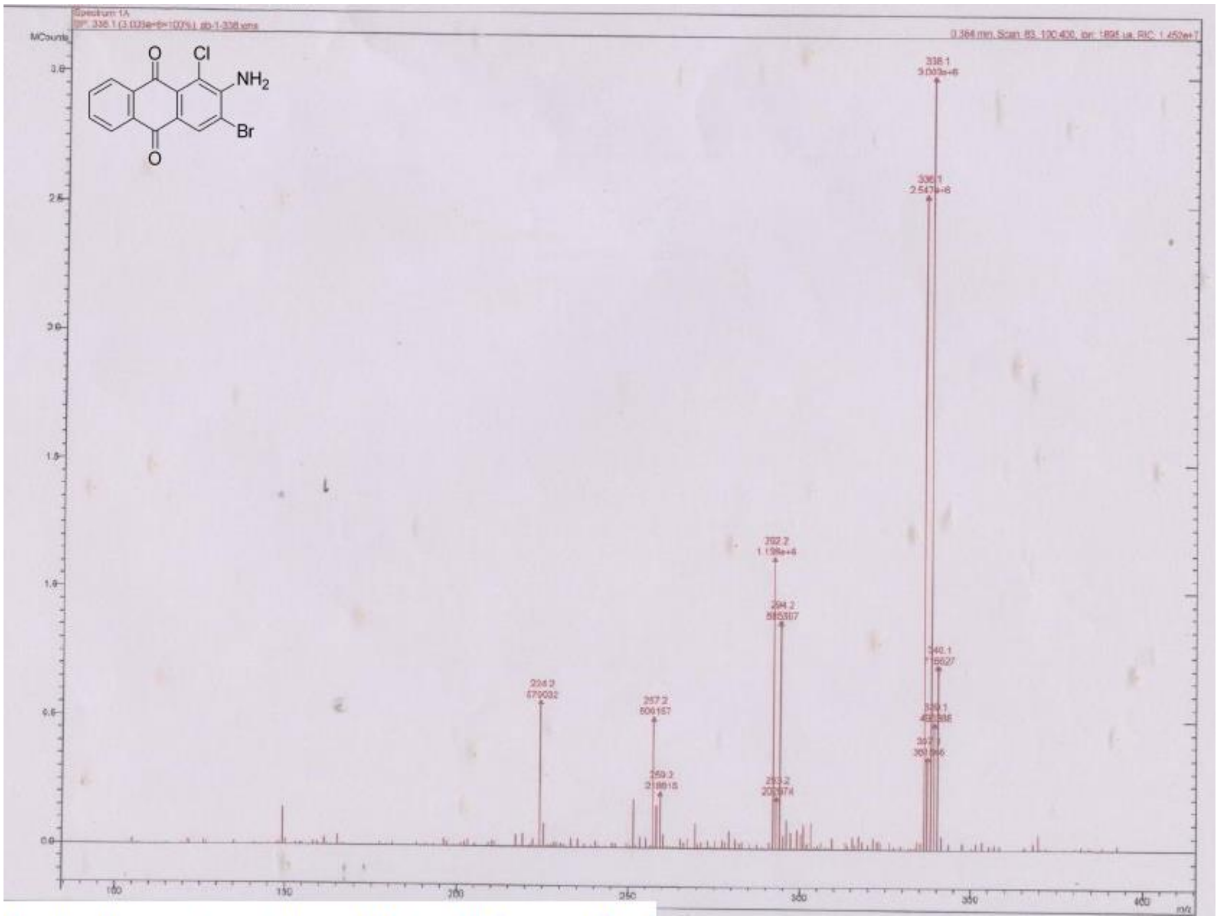

3) 1-Amino-2, 4-dibromo-5-chloroanthracene-9,10-dione (2c)

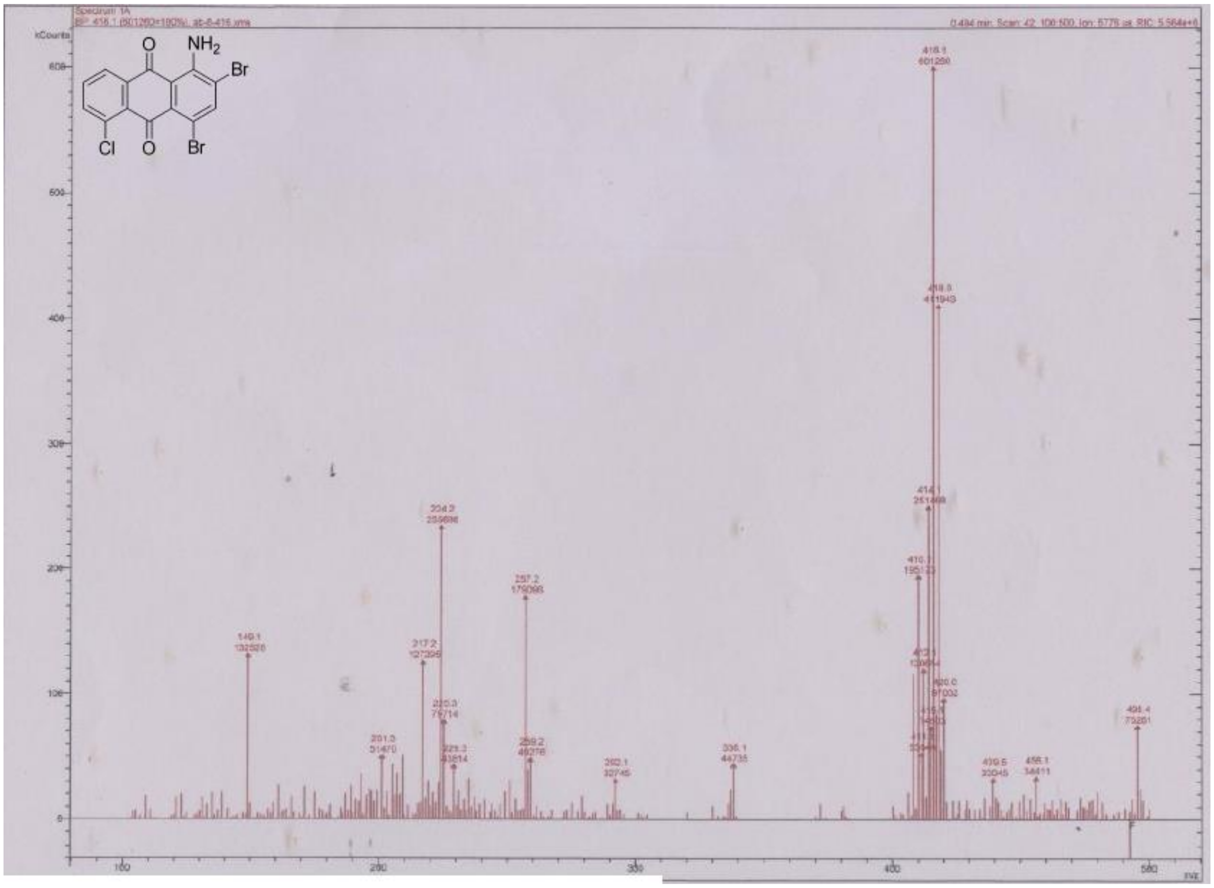

4) 2-Amino-1,3-dibromoanthracene-9,10-dione (2d)

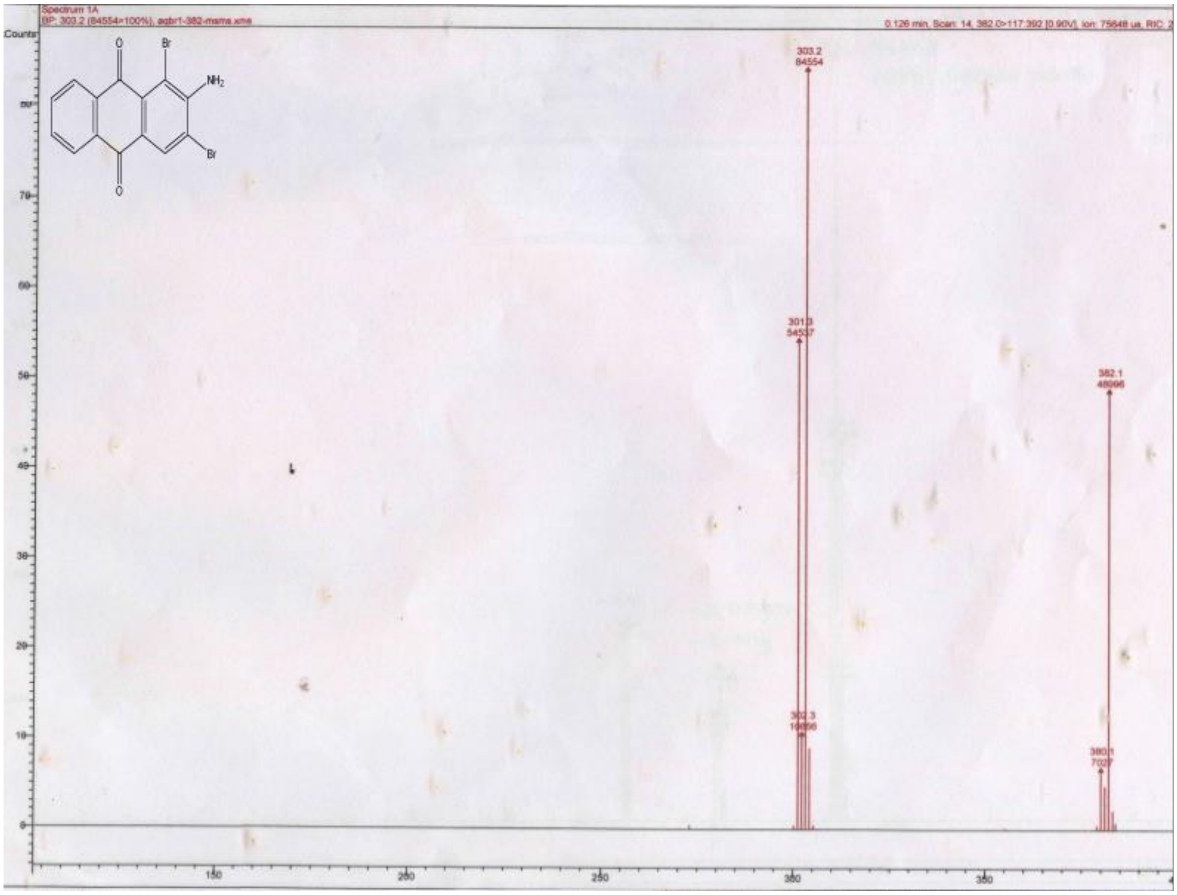

5) 1-Amino-2,4-dibromo-5-(phenylamino)anthracene-9,10-dione (2e)

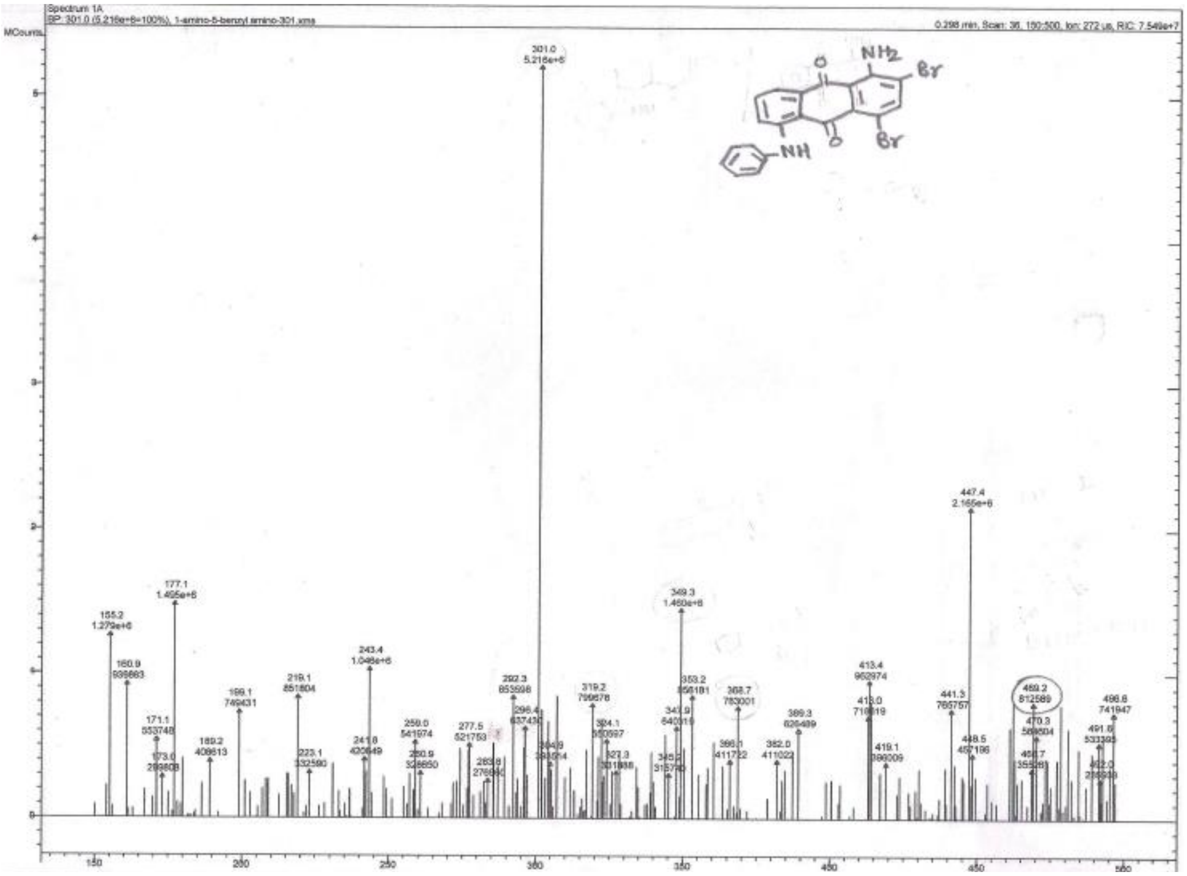

6) **1,5-Diamino-2,4,6,8-tetrabromoanthracene-9,10-dione (2f)**

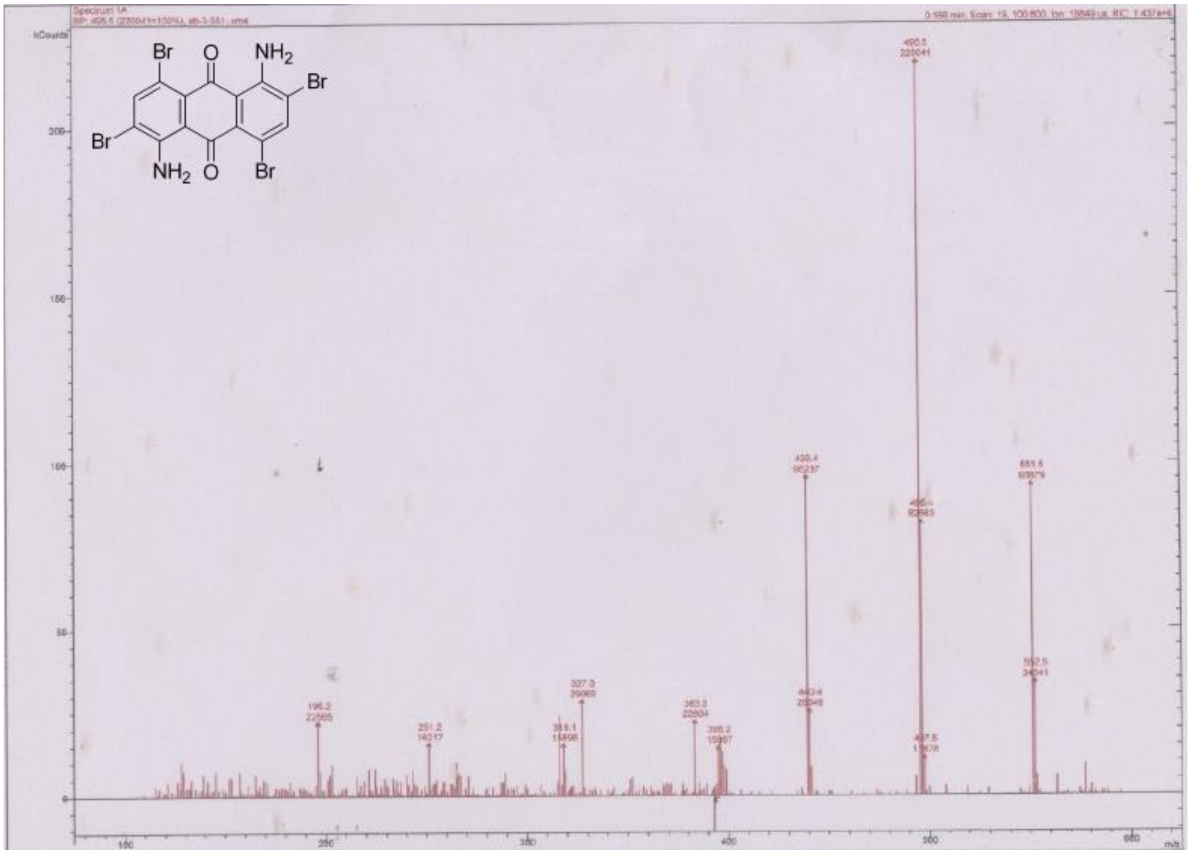

7) 1-Amino-2-bromo-4-methoxyanthracene-9,10-dione (2g)

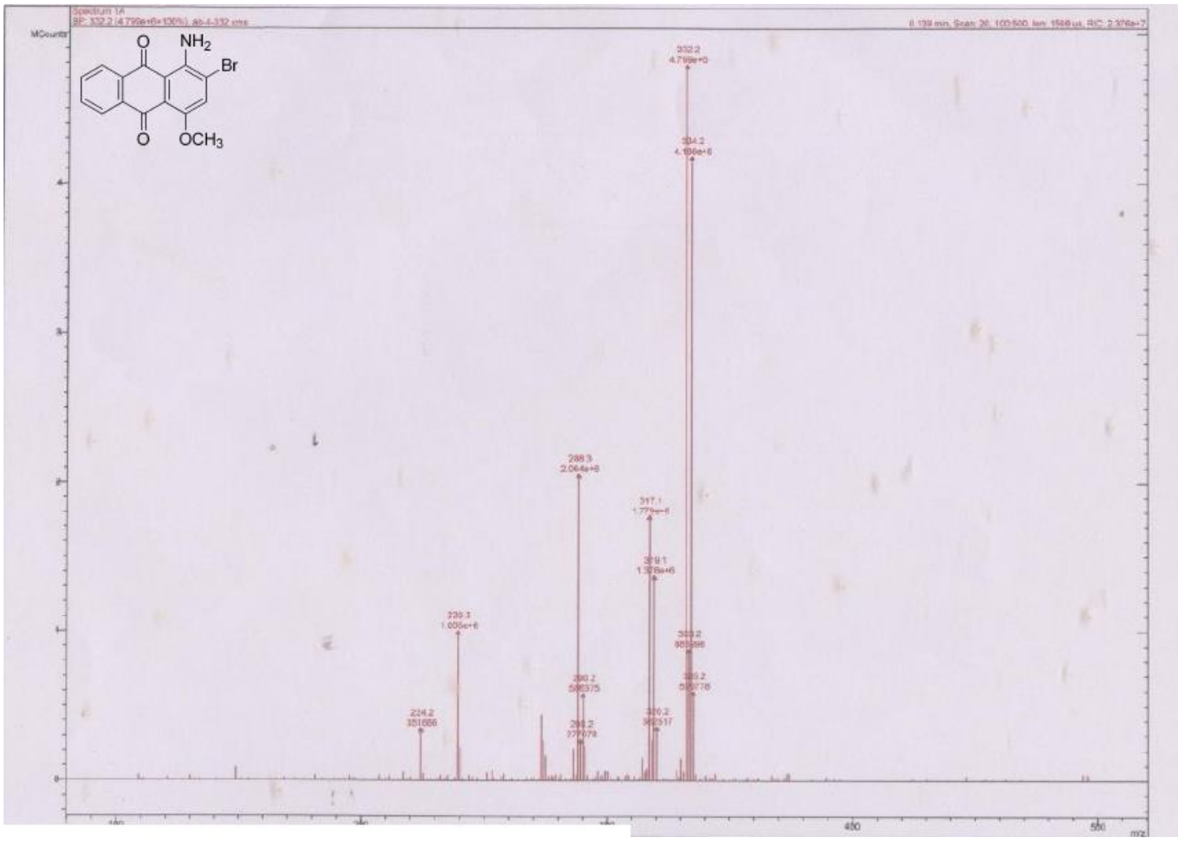

8) *N*-(4-amino-3-bromo-9,10-dioxo-9,10-dihydroanthracen-1-yl)benzamide (2h)

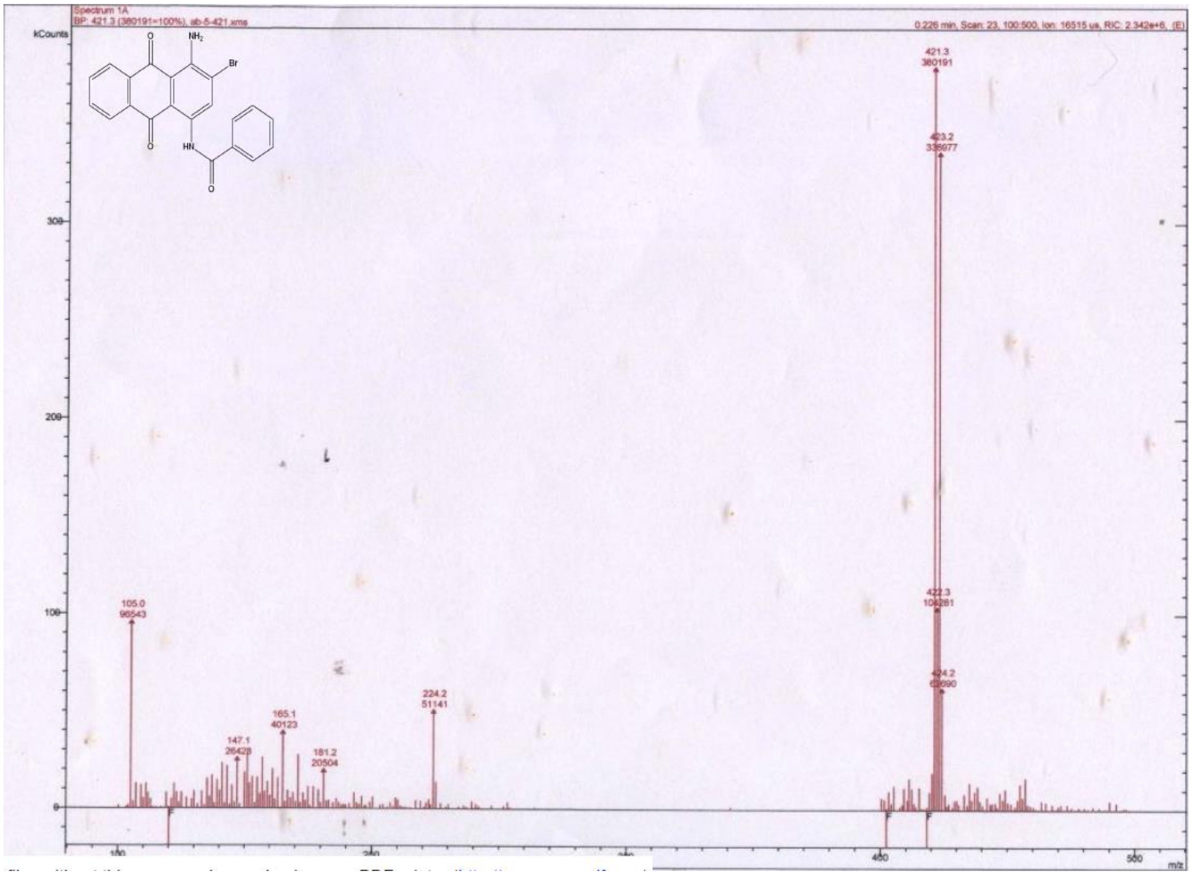

9) 2,4-Dibromo-1-(methylamino)anthracene-9,10-dione (2i)

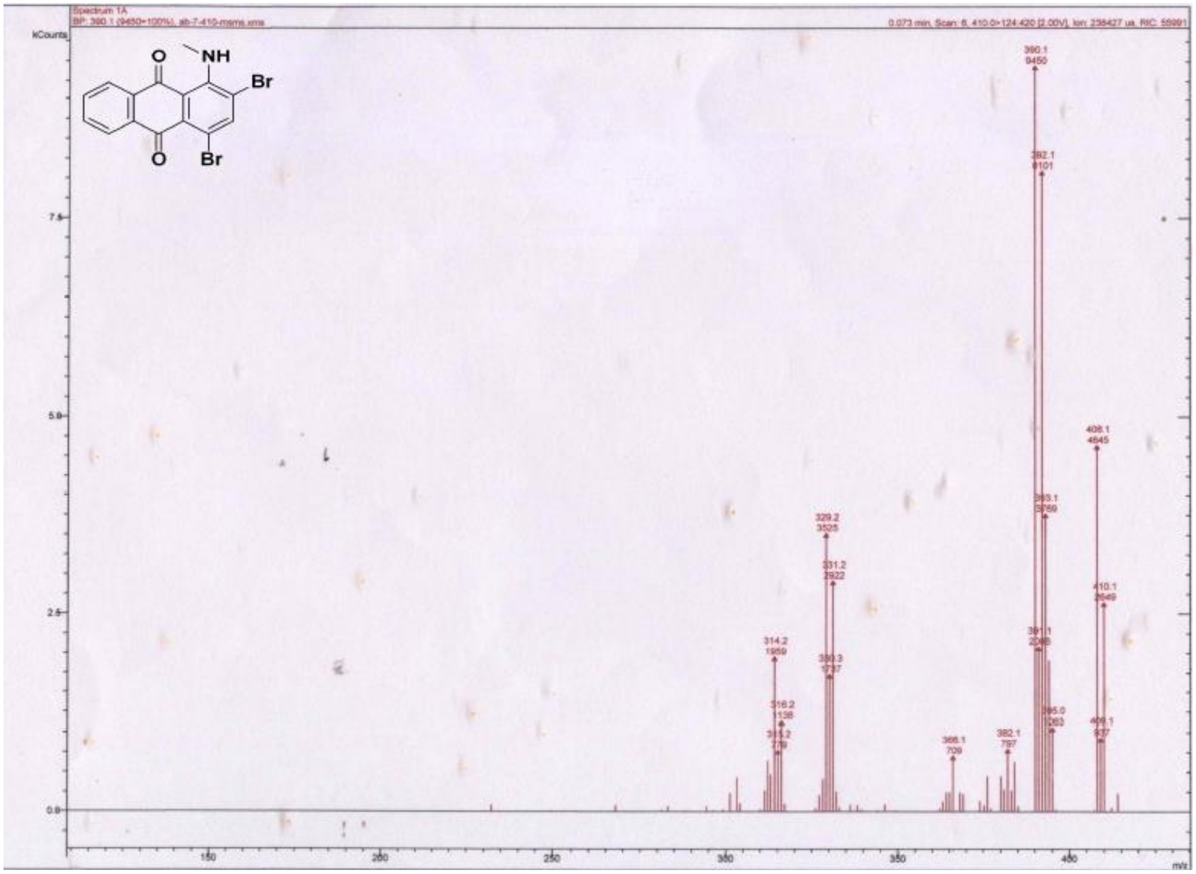

10) **2,4-Dibromo-1-(ethylamino)anthracene-9,10-dione (2j)**

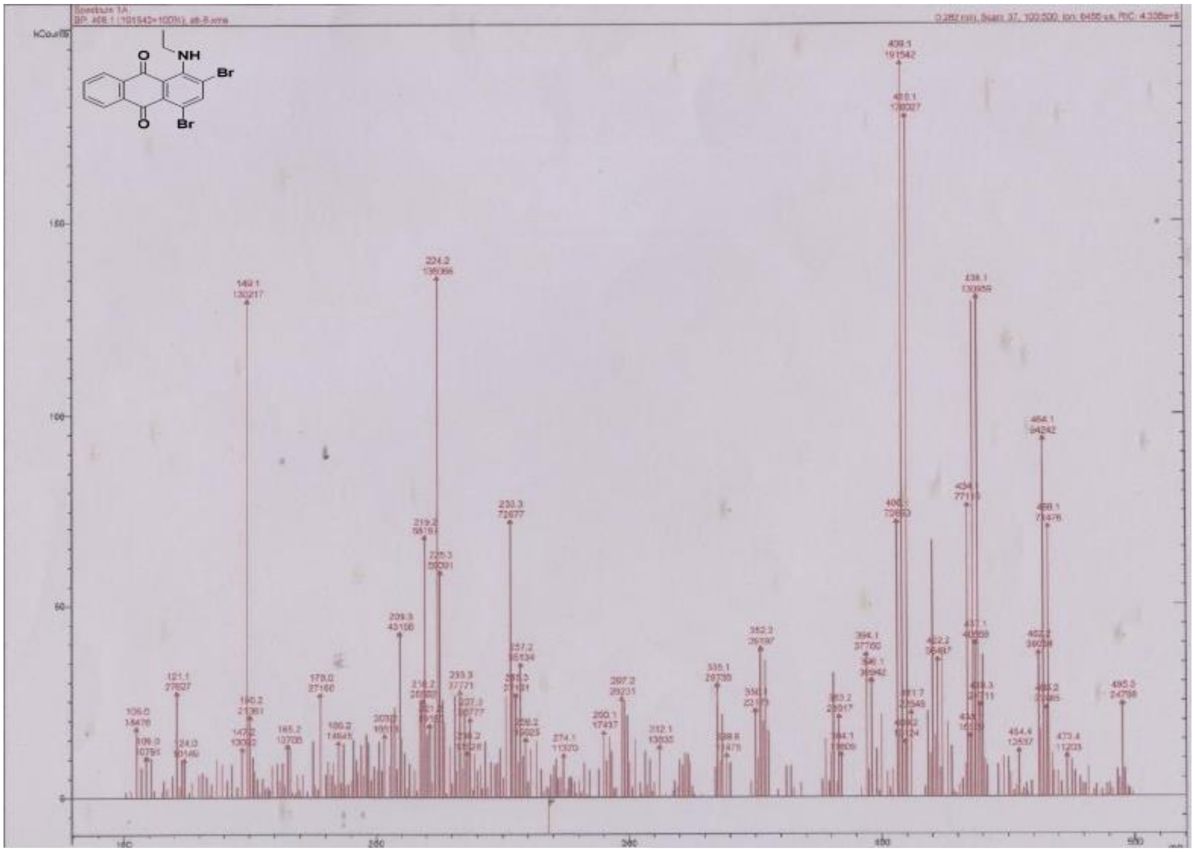

11) 3-Bromo-7*H*-benzo[*d*,*e*]anthracen-7-one (2k)

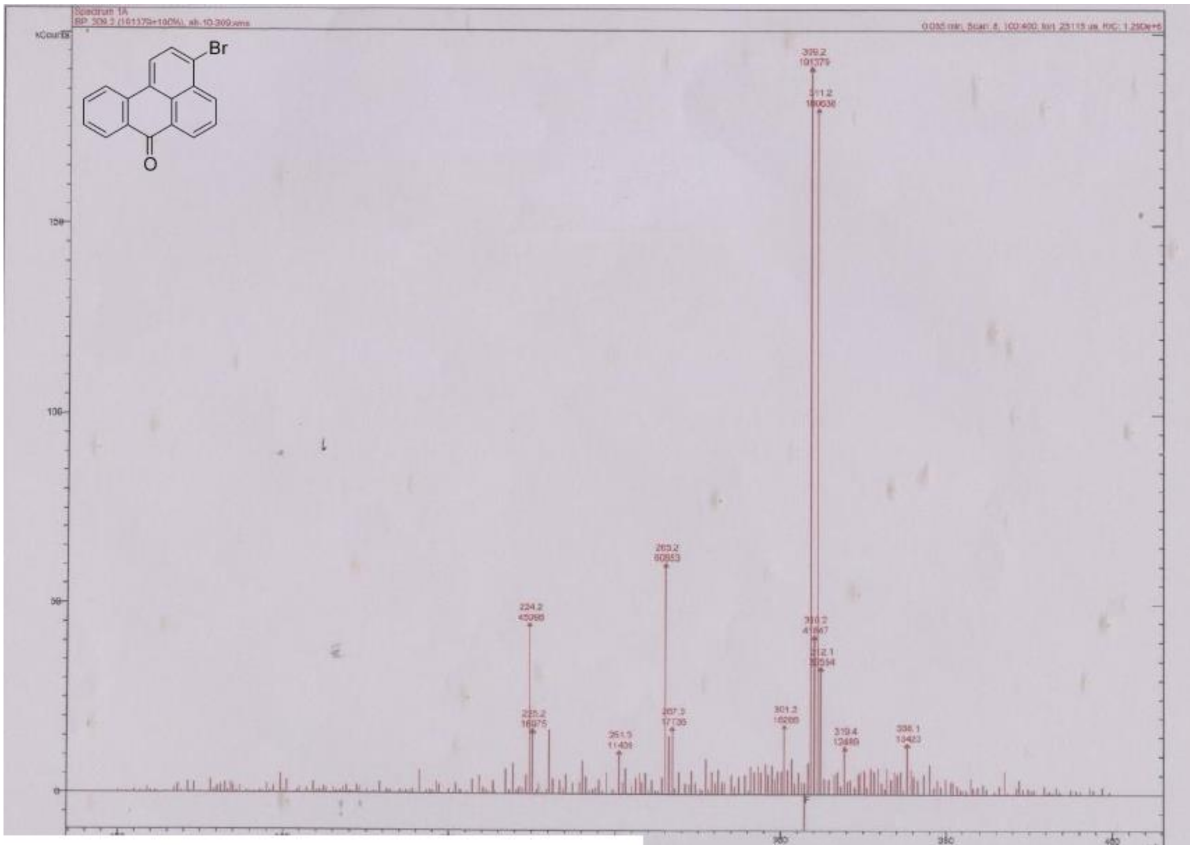

^{1}H NMR Spectra

1) **1-Amino-2,4-dibromoanthracene-9,10-dione (2a)**

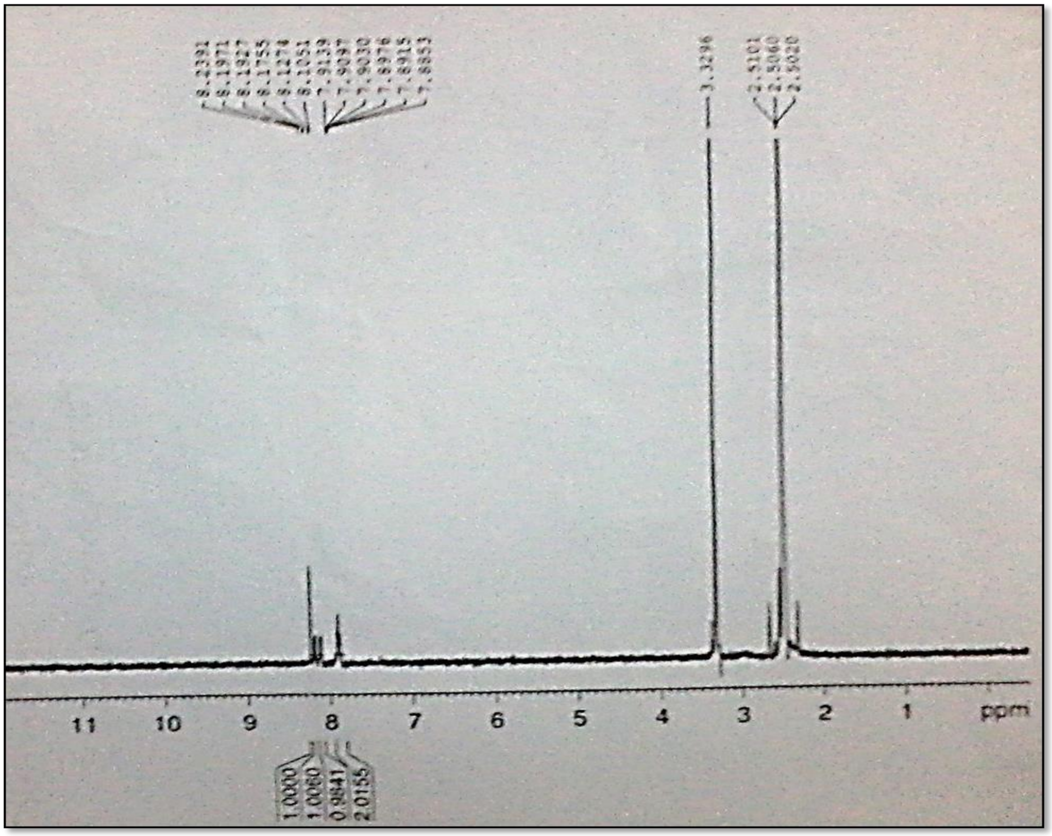

2) 2-Amino-3-bromo-1-chloroanthracene-9,10-dione (2b)

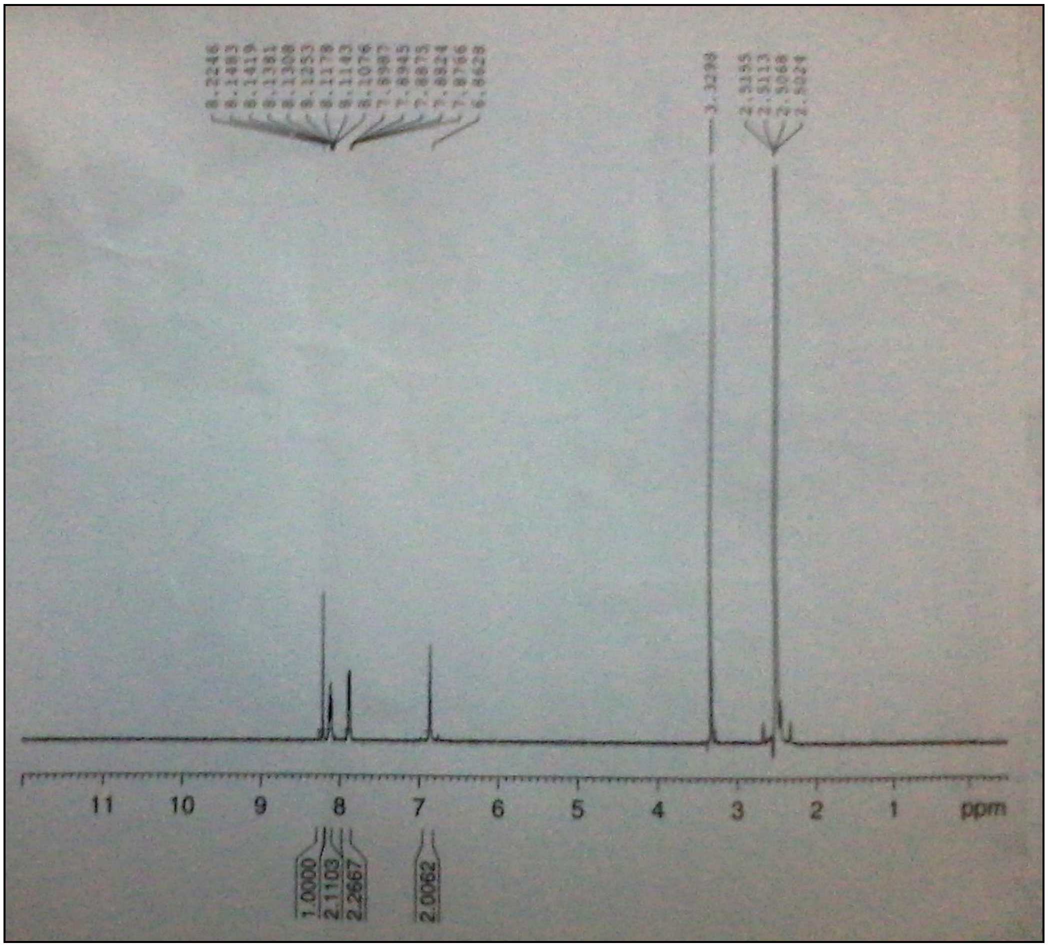

3) 1-Amino-5-chloro-2,4-diaminoanthracene-9,10-dione (2c)

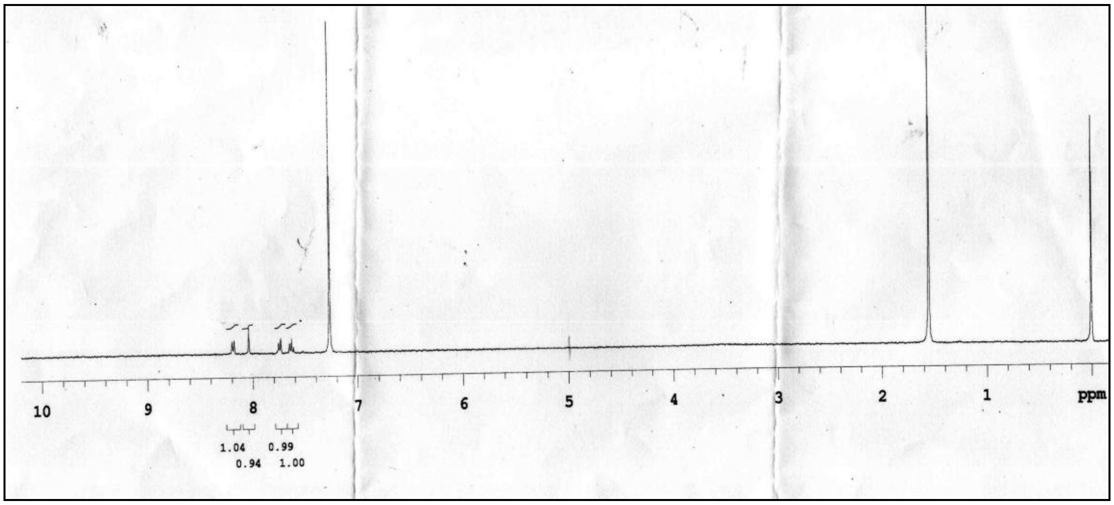

4) 2-Amino-1,3-dibromoanthracene-9,10-dione (2d)

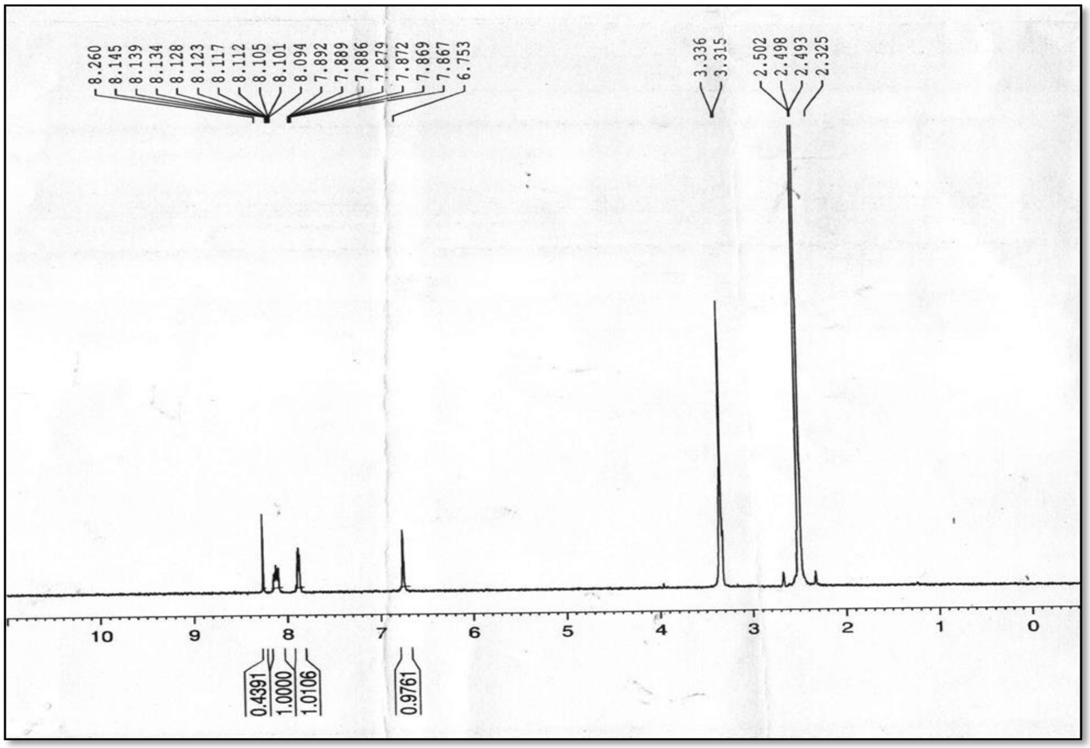

5) 1-Amino-2,4-dibromo-5-(phenylamino)anthracene-9,10-dione (2e)

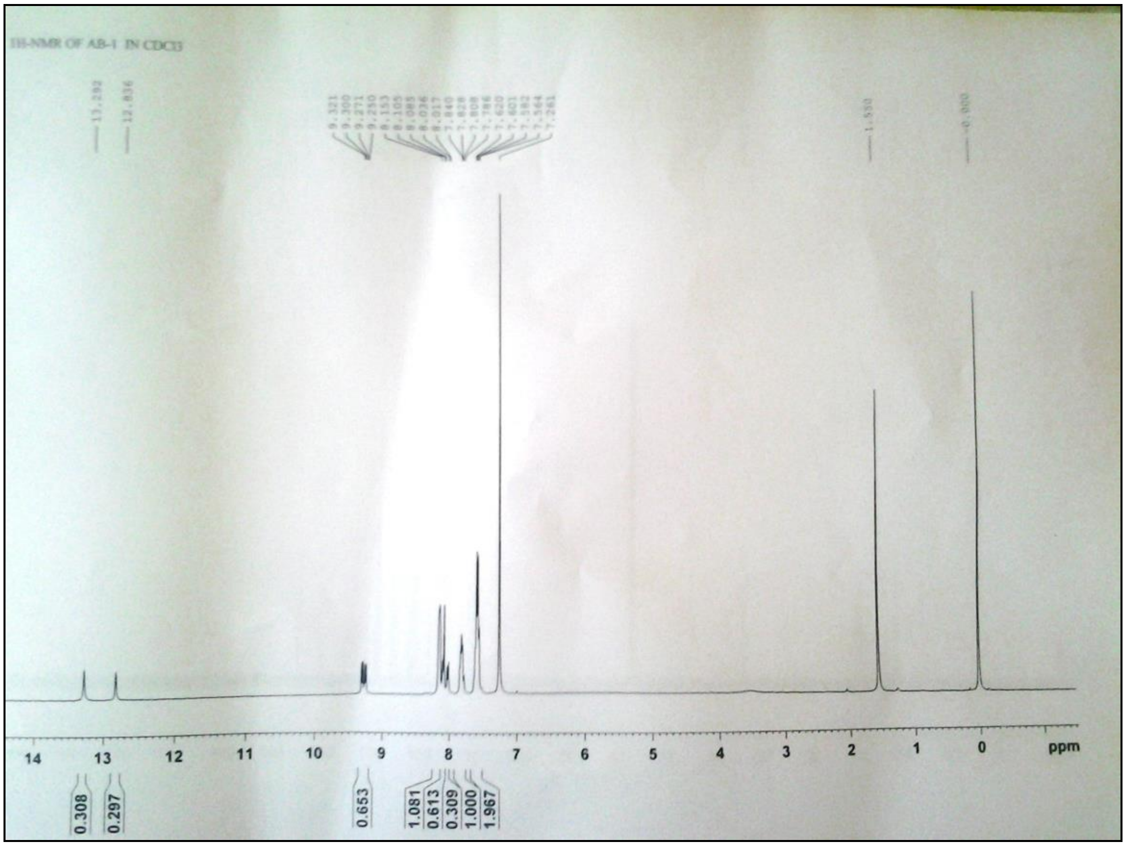

6) 1,5-Diamino-2,4,6,8-tetrabromoanthracene-9,10-dione (2f)

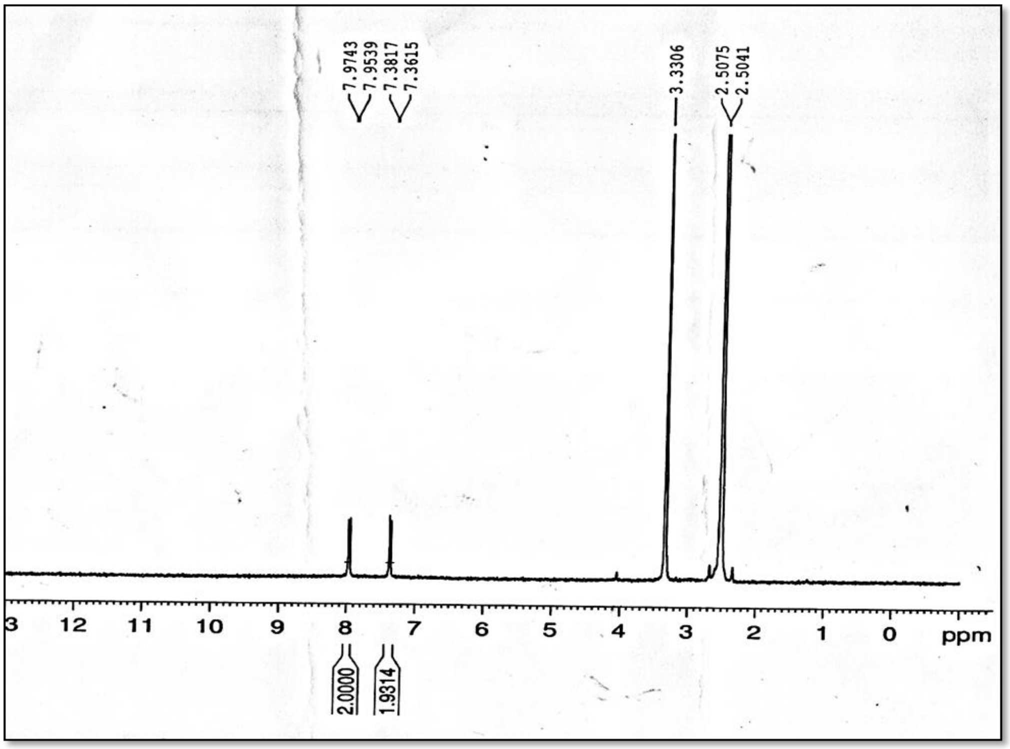

7) **1-Amino-2-bromo-4-methoxyanthracene-9,10-dione (2g)**

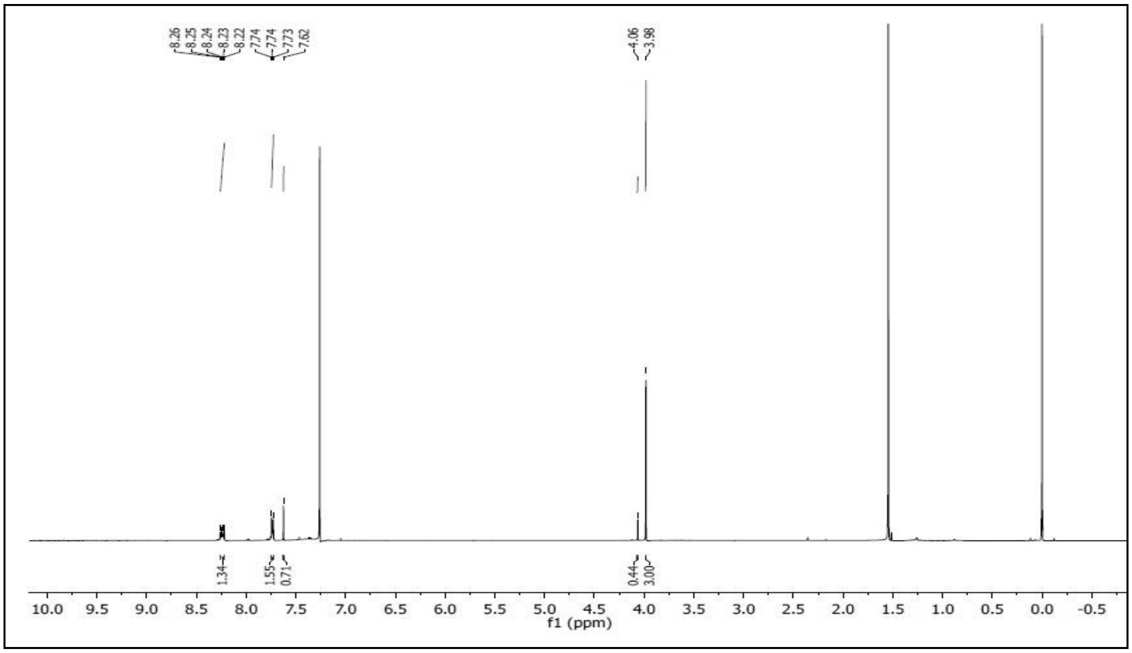

8) N-(4-amino-3-bromo-9,10-dioxo-9,10-dihydroanthracen-1-yl)benzamide (2h)

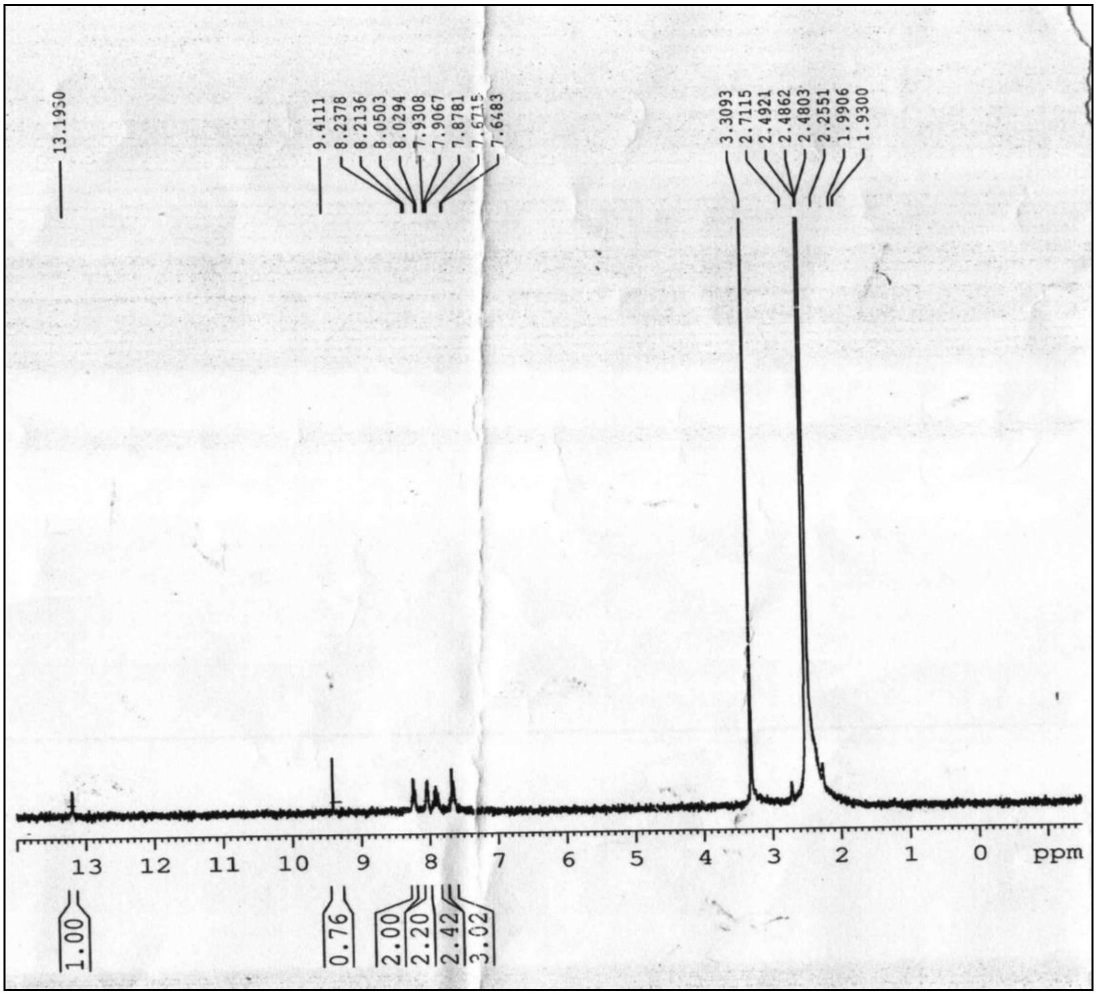

9) 2,4-Dibromo-1-(methylamino)anthracene-9,10-dione (2i)

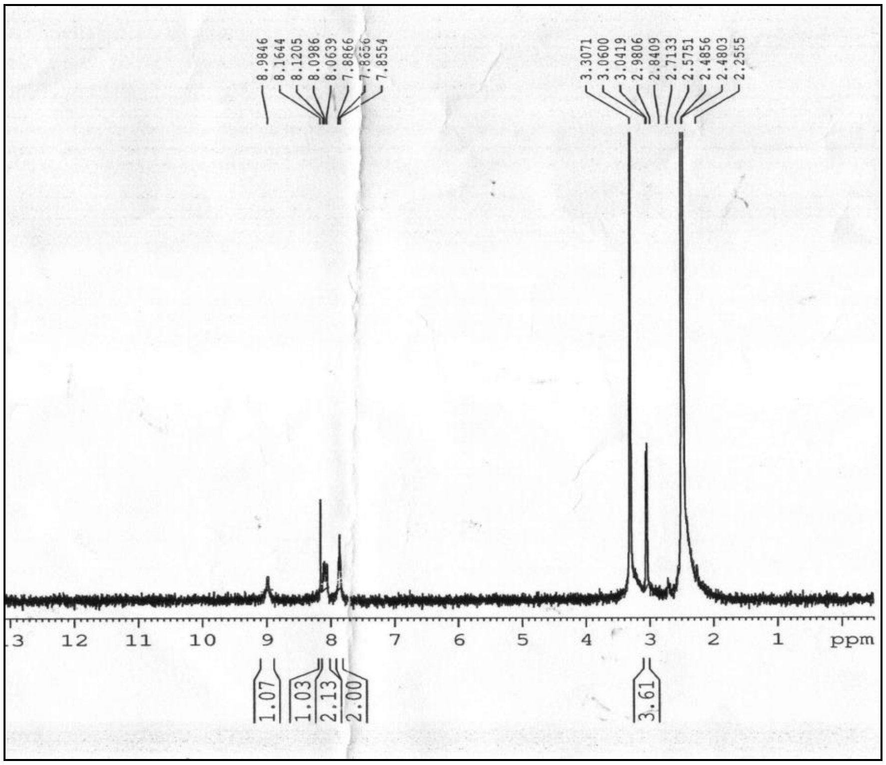

10) 2,4-Dibromo-1-(ethylamino)anthracene-9,10-dione (2j)

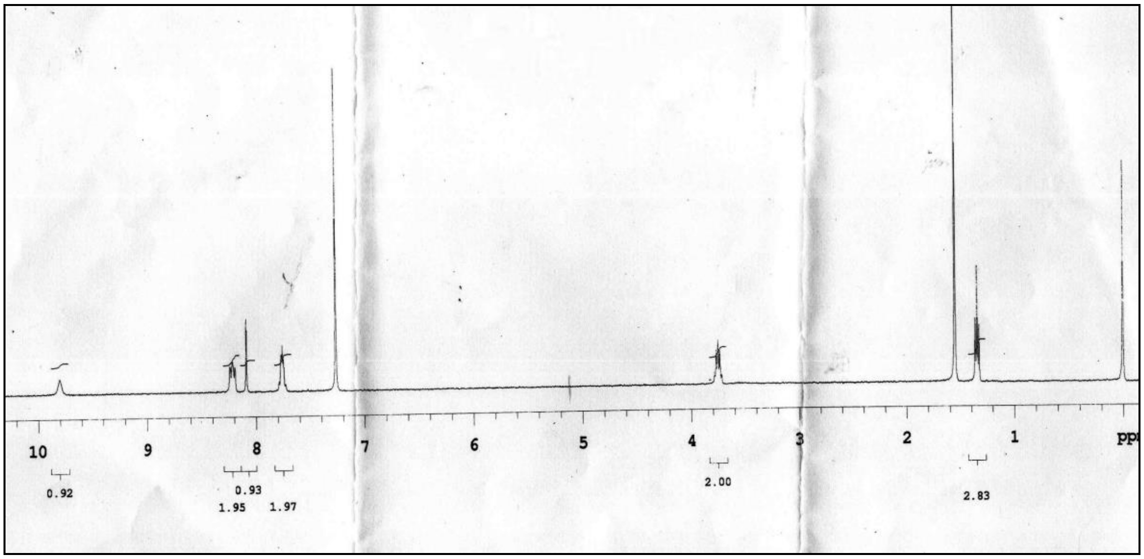

11) 3-Bromo-7H-benzo[d,e]anthracen-7-one (2k)

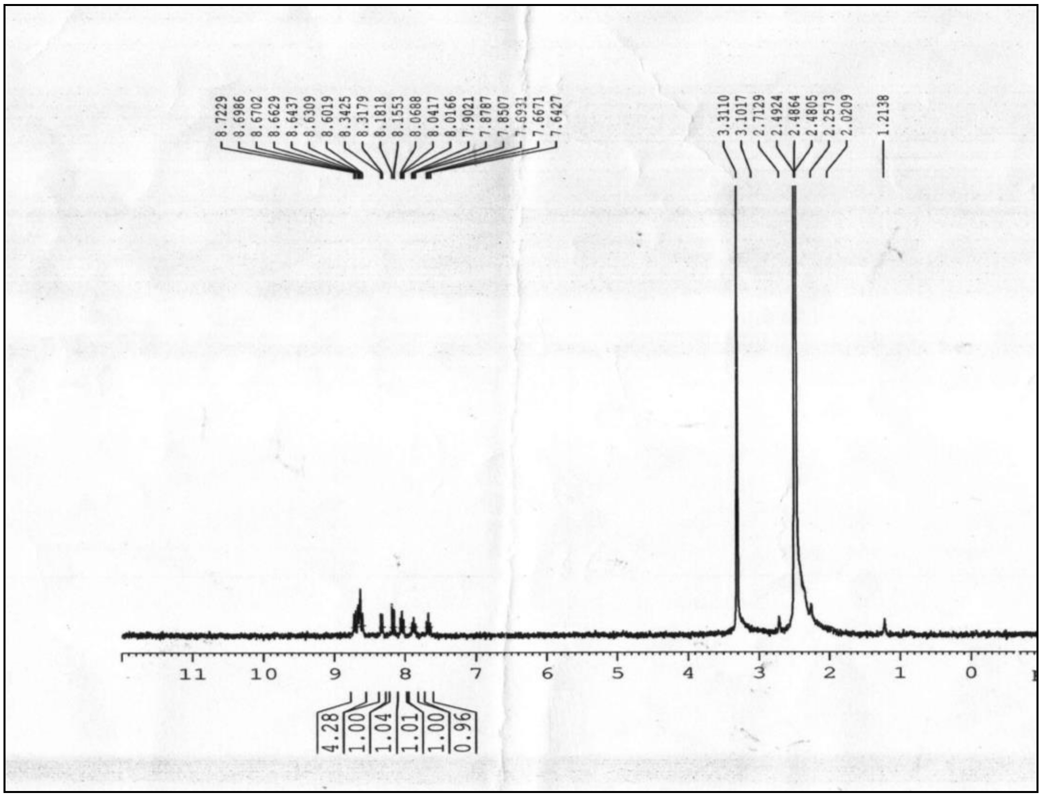

DSC analysis of nonanebis(peroxoic acid)

- **Instrument**: DSC Q100 V9.9 Build 303 (Universal V4.5A TA Instrument)
- **Operation conditions**:
  a. Ramp 10.00 °C/min to 300.00 °C
  b. Gas: Nitrogen
  c. Flow rate: 50.0 ml/min
  d. Sample weight taken: 1.3 mg
- **DSC graph of nonanebis(peroxoic acid):**

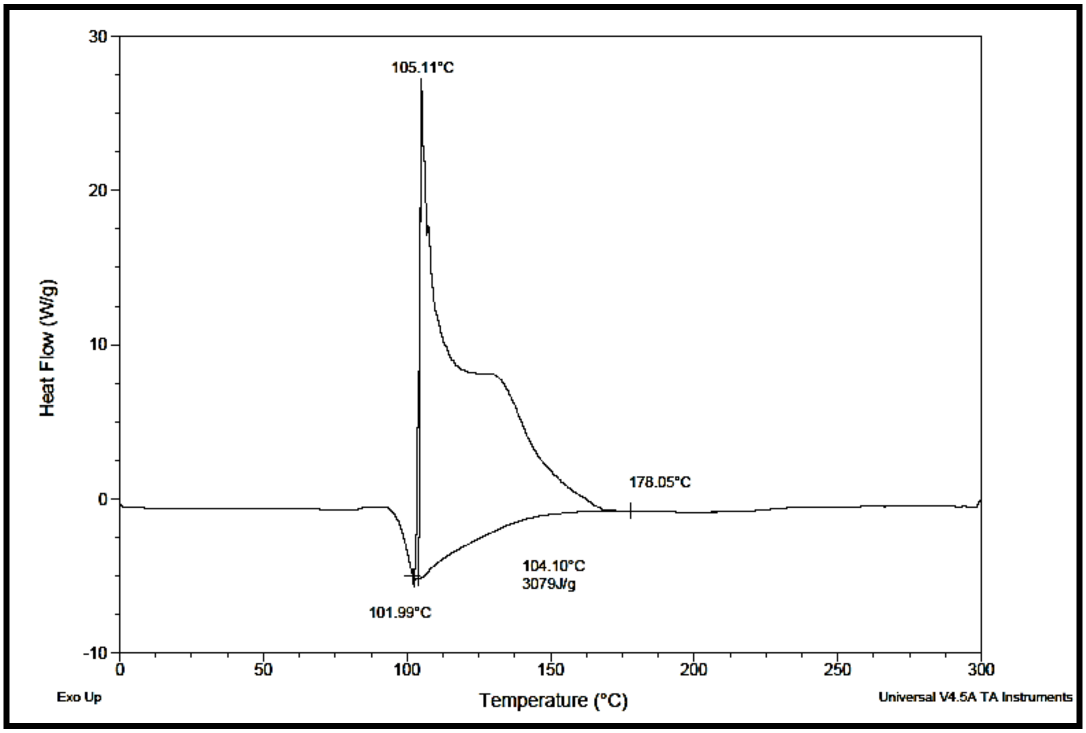

- **According to Yoshida's correlation equation:** [1]

1. **Shock Sensitivity (SS)** = [log (Q_{dsc}) − 0.72] [log (T_{dsc} – 25) – 0.98]
2. **Explosion Propagation (EP)** = [log (Q_{dsc}) − 0.38] [log (T_{dsc} – 25) – 1.67]

Where, $\boldsymbol{Q_{dsc}}$ = Energy of the exotherm in calories/g

$\boldsymbol{T_{dsc}}$ = The onset temperature of the exotherm in °C

➢ **Calculation**:

1. **The values of $\boldsymbol{T_{dsc}}$ and $\boldsymbol{Q_{dsc}}$ obtained from DSC graph**

    a. $\boldsymbol{T_{dsc}}$ = 104.10 °C

    b. $\boldsymbol{Q_{dsc}}$ = 3079 J/g = 0.0007354 calories/g

2. **<u>Shock Sensitivity (SS)</u>**

$$\textbf{SS} = [\log (Q_{dsc}) - 0.72]\,[\log (T_{dsc} - 25) - 0.98]$$
$$= [\log (0.0007354) - 0.72]\,[\log (104.1 - 25) - 0.98]$$
$$= [-3.8534]\,[0.9181]$$
$$= \mathbf{-\,3.5381}$$

3. **<u>Explosion Propagation (EP)</u>**

$$\textbf{EP} = [\log (Q_{dsc}) - 0.38]\,[\log (T_{dsc} - 25) - 1.67]$$
$$= [\log (0.0007354) - 0.38]\,[\log (104.1 - 25) - 1.67]$$
$$= [-3.6134]\,[0.2281]$$
$$= \mathbf{-\,0.8016}$$

➢ **<u>Conclusion</u>**:

"*According to this equation, if the value for Shock sensitivity* ***(SS)*** *or Explosion Propagation* ***(EP)*** *is ≥0.00, then the material is predicated to be shock sensitive or demonstrate explosive propagating properties, respectively*."

From the above calculation it was clear that, the nonanebis(peroxoic acid) is non-shock sensitive as well as it does not exhibit explosive propagation properties.

Details of HPLC analysis

Sr. No.	Parameters	Description
1.	Instrument	Jasco 2000
2.	Column	HiQ Sil C18HS, size : 4.6 mm × 250 mm.
3.	Mobile Phase	90% acetonitrile + 10% methanol
4.	Flow rate	1 ml/min
5.	Wavelength	235 nm
6.	Detector	UV detector
7.	Sample dilution	All samples are dissolved in mobile phase
8.	Sample injected	5 microliters

Note: All solvents used as HPLC grade

Detection of active oxygen content

Preparation of solutions:

1. 0.1 M $Na_2S_2O_3{\cdot}5H_2O_2$: Dissolve 25 g sodium thiosulfate in 1 L boiled distilled water.
2. 50% Potassium Iodide: 50 g potassium iodide in 50 mL distilled water.
3. 10% (v/v) sulfuric acid: Take 10 mL 98% sulfuric acid and dilute it up to 100 mL with distilled water.
4. Starch solution: Take 0.1 g starch and dissolve it in 100 mL distilled water. Heat the above solution till it boil. Then cool this solution to rt (room temperature)
5. Solvent for water insoluble peracids : (Acetic acid/chloroform) (3:2)

Process:

1. In 250 mL iodine flask, take 0.15 to 4 g sample of peroxy compound.
2. Dissolve it in 20 mL (acetic acid + chloroform) solvent.
3. Add 2 mL 50% potassium iodide solution.
4. Stopper immediately flask, and keep for 5 min.
5. Add 50 mL distilled water.
6. Titrate against 0.1 M sodium thiosulfate solution.
7. Add starch indicator towards endpoint.
8. End point colorless to blue color.

Calculation:

$$\%\ \text{Active } O_2 \text{ content} = \frac{(\text{Burette reading})\ (\text{Normality of } Na_2S_2O_3)\ (0.008)\ (100)}{(\text{Sample weight})}$$

Reference

1. Richard, K.; Lim, M.; Hachmeister, Z. *Process Safety News*, **2010,** *17*, 3–4. https://www.fauske.com/sites/default/files/ShockExplosionandFrictionHazards-IdentificationandMitigation.pdf
